# Effectiveness of an Advance Care Planning Intervention in Adults Receiving Dialysis and Their Families: A Cluster Randomized Clinical Trial

**DOI:** 10.1001/jamanetworkopen.2023.51511

**Published:** 2024-01-30

**Authors:** Mi-Kyung Song, Amita Manatunga, Laura Plantinga, Maureen Metzger, Abhijit V. Kshirsagar, Janice Lea, Emaad M. Abdel-Rahman, Manisha Jhamb, Emily Wu, Jacob Englert, Sandra E. Ward

**Affiliations:** 1 Center for Nursing Excellence in Palliative Care, Nell Hodgson Woodruff School of Nursing, Emory University, Atlanta, Georgia; 2 Rollins School of Public Health, Emory University, Atlanta, Georgia; 3 Division of Rheumatology, Department of Medicine, University of California, San Francisco; 4 Division of Nephrology, Department of Medicine, University of California, San Francisco; 5 School of Nursing, University of Virginia, Charlottesville; 6 UNC Kidney Center, University of North Carolina at Chapel Hill School of Medicine; 7 Division of Nephrology and Hypertension, University of North Carolina at Chapel Hill School of Medicine; 8 Division of Renal Medicine, Emory University School of Medicine, Emory University, Atlanta, Georgia; 9 Division of Nephrology, University of Virginia School of Medicine, University of Virginia, Charlottesville; 10 Division of Renal-Electrolyte, Department of Medicine, University of Pittsburgh School of Medicine, Pittsburgh, Pennsylvania; 11 School of Nursing, University of Wisconsin–Madison

## Abstract

**Importance:**

Evidence of effectiveness of advance care planning (ACP) strategies for patients receiving dialysis and their families is needed.

**Objectives:**

To test the effectiveness of an ACP intervention to prepare patients and their surrogates for end-of-life (EOL) decision-making and to improve surrogate bereavement outcomes.

**Design, Setting, and Participants:**

This cluster randomized clinical trial, An Effectiveness-Implementation Trial of SPIRIT (Sharing Patients’ Illness Representations to Increase Trust) in ESRD, was conducted from December 2017 to March 2023 and included 42 dialysis clinics in 5 US states (Georgia, New Mexico, North Carolina, Pennsylvania, and Virginia) randomized to provide intervention or usual care. Recruitment was from February 15, 2018, to January 31, 2022, and patient-surrogate dyads were followed up for 21 months (until January 17, 2023) or until patient death.

**Intervention:**

Each clinic selected 1 or 2 health care workers (eg, nurse practitioner, registered nurse, or social worker) to conduct 45- to 60-minute ACP discussions with dyads in the clinic or remotely. After March 13, 2020 (commencement of the COVID-19 emergency declaration), all discussions were conducted remotely. An ACP summary was placed in patients’ medical records.

**Main Outcomes and Measures:**

The primary, 2-week preparedness outcomes were dyad congruence on EOL goals of care, patient decisional conflict, surrogate decision-making confidence, and a composite of dyad congruence and surrogate decision-making confidence. Secondary bereavement outcomes were anxiety, depression, and posttraumatic distress 3 months after patient death. To adjust for COVID-19 pandemic effects on bereavement outcomes, a variable to indicate the timing of baseline and 3-month assessment relative to the COVID-19 emergency declaration was created.

**Results:**

Of the 426 dyads enrolled, 231 were in the intervention clinics, and 195 were in the control clinics. Among all dyads, the mean (SD) patient age was 61.9 (12.7) years, and the mean (SD) surrogate age was 53.7 (15.4) years. At 2 weeks, after adjusting for baseline values, dyad congruence (odds ratio [OR], 1.61; 95% CI, 1.12-2.31; *P* = .001), decisional conflict scores (β, −0.10; 95% CI, −0.13 to −0.07; *P* < .001), and the composite (OR, 1.57; 95% CI, 1.06-2.34; *P* = .03) were higher in the intervention group than in the control group. Surrogate decision-making confidence was similar between groups (β, 0.06; 95% CI, −0.01 to 0.13; *P* = .12). Among 77 bereaved surrogates, after adjusting for baseline values and assessment timing, intervention group anxiety was lower than control group anxiety (β, −1.55; 95% CI, −3.08 to −0.01; *P* = .05); however, depression (β, −0.18; 95% CI, −2.09 to 1.73; *P* = .84) and posttraumatic distress (β, −0.96; 95% CI, −7.39 to 5.46; *P* = .75) were similar.

**Conclusions and Relevance:**

In this randomized clinical trial, the ACP intervention implemented by health care workers at dialysis centers improved preparation for EOL decision-making but showed mixed effectiveness on bereavement outcomes. The ACP intervention implemented in dialysis centers may be an effective strategy to the dyad preparation for end-of-life care as opposed to the current focus on advance directives.

**Trial Registration:**

ClinicalTrials.gov Identifier: NCT03138564

## Key Points

**Question:**

Can an advance care planning intervention implemented by health care workers at dialysis centers affect patient and surrogate preparedness for end-of-life decision-making?

**Findings:**

In this randomized clinical trial of 426 dyads of patients receiving dialysis and their surrogate decision-makers, the intervention group, compared with a usual care control group, was superior in dyad congruence on end-of-life care goals, patient decisional conflict, and a composite of dyad congruence and surrogate decision-making confidence. Among surrogates with bereavement outcomes, intervention group anxiety was lower than in the control group, but depression and posttraumatic distress were similar.

**Meaning:**

Results of this trial demonstrated that the advance care planning intervention by health care workers at dialysis centers improved patient and surrogate preparation for end-of-life decision-making, which may in turn reduce surrogates’ distress after the patient’s death.

## Teaser

This randomized clinical trial assesses the effectiveness of an intervention in which health care workers at dialysis centers discuss advance care planning strategies of patients receiving dialysis and their surrogates compared with patients receiving usual care at US dialysis centers.

## Introduction

Kidney failure is a family of multiple chronic conditions, commonly including diabetes and hypertension. Patients receiving dialysis have a mortality rate 6 to 8 times greater than that of the general public.^1^ Compared with older adults (aged ≥65 years) with congestive heart failure or cancer, those undergoing dialysis are more likely to be hospitalized, receive intensive procedures in the last 30 days of life, experience lower quality end-of-life (EOL) care, and die in a hospital,^2^ which are levels of treatment intensity that may be incongruent with their values.^3,4,5^

For decades, advance care planning (ACP) has been promoted as a way to ensure goal-concordant EOL care.^6,7^ However, a large body of literature has revealed that aligning care with a patient’s preferences is extremely difficult.^8,9^ End-of-life care is often driven by a more-is-better approach that is reinforced by a chain of social, economic, and bureaucratic forces favoring aggressive intervention.^10^ Research has shown that a belief in preserving life at all costs may lead to the decision to start dialysis, which, once started, is difficult to stop.^11,12^ For patients receiving dialysis and their family members, defaulting to aggressive EOL care may be predictable if they have had no opportunity to carefully prepare for EOL decision-making.

Advance care planning, focused narrowly on completing advance directives (ADs) in an attempt to steer patients with serious illness away from aggressive treatment, has had limited success.^13,14,15^ Furthermore, studies of interventions to support family decision-makers near the EOL (ie, in intensive care units)^16,17,18^ have not succeeded in improving bereavement distress, suggesting that intervening during this period of emotional turmoil may be too late. These shortcomings have led some to argue for abandoning efforts to improve ACP altogether.^19^

In contrast to focusing on ADs and waiting until EOL is near, our ACP intervention, Sharing the Patient’s Illness Representations to Increase Trust (SPIRIT), was designed to help patients receiving dialysis and their surrogate decision-makers (dyads) jointly prepare for EOL decision-making and improve surrogates’ bereavement outcomes.^20,21^ The SPIRIT intervention has been tested over multiple pilot and efficacy randomized clinical trials.^22,23,24,25^ In a full-scale efficacy trial,^24^ SPIRIT was associated with improvements in preparedness outcomes and surrogates’ bereavement outcomes. In this study, we examined the effectiveness of SPIRIT on preparedness outcomes for patients undergoing dialysis and their surrogates and surrogates’ bereavement outcomes.

## Methods

### Design

This study (An Effectiveness-Implementation Trial of SPIRIT in ESRD) was a 2-group, pragmatic cluster randomized clinical trial with measures of patient and surrogate preparedness at baseline and 2 weeks later for the control group or 2 weeks after the SPIRIT intervention and with measures of surrogate bereavement outcomes at baseline and 3 months after a patient’s death (see the trial protocol in Supplement 1). This study was more pragmatic than explanatory in the pragmatic-explanatory continuum because it included diverse patient populations, heterogeneous settings, and few inclusion and exclusion criteria, and the intervention was conducted by health care workers at the dialysis clinic, not by study interventionists, with minimal control. This study followed the Consolidated Standards of Reporting Trials (CONSORT) reporting guideline. We limited outcome measurements to baseline and a 1-time follow-up for effectiveness estimation. Data were collected by telephone, outcome assessors were blinded to group assignment, and the protocol was approved by each study site’s institutional review board. Patients provided written informed consent, and subsequent to patient consent, recruiters telephoned surrogates to obtain verbal consent.

### Dialysis Clinics and Randomization

Forty-two freestanding dialysis clinics were randomized (23 for the intervention group and 19 for the usual care control group). Each clinic selected 1 or 2 health care workers (eg, nurse practitioner, registered nurse, or social worker), or SPIRIT champions, to conduct SPIRIT sessions. Clinics were stratified by census size: small (≤52), medium (53-105), and large (≥106) and randomized in permuted blocks of 2 or 4 within strata nested in state, with a condition that clinics covered by the same SPIRIT champion would be allocated to the same group. When a site was ready to begin the study, the coordinating center informed the site investigator of the group assignment so that group-specific clinic orientation and champion training could begin.

### Participants

The study was conducted from December 2017 to March 2023. Recruitment began February 15, 2018, and ended January 31, 2022. The protocol was modified in February 2019 to increase the number of surrogates for bereavement outcomes: dyads that reached 9 months (the original end of participation) were invited to remain in the study for an additional 12 months. Between March and September 2020, all research activities, except telephone-based data collection, were halted upon the COVID-19 emergency declaration. Patient-surrogate dyads were followed up for 21 months (until January 17, 2023) or until patient death.

Patient eligibility criteria were age 18 years or older and the ability to understand and speak English. Patients without a surrogate, too ill or cognitively impaired based on clinicians’ judgment, or enrolled in hospice were excluded. Surrogate eligibility criteria were age 18 years or older, the ability to understand and speak English, and designation as the surrogate by the patient. Each clinic’s champion generated a list of eligible patients quarterly and obtained approval from the treating nephrologist before assessing the patient’s willingness to meet with a study recruiter. Race and ethnicity categories included Hispanic Black, Hispanic White, non-Hispanic Black, non-Hispanic White, and other (Asian, Native American, and more than 1 race and/or ethnicity). Classification was based on a self-reported demographic questionnaire completed by each participant and was included in the study because of the potential influence of race and ethnicity on study outcomes.

### Intervention

There were 2 face-to-face sessions with a patient-surrogate dyad. Champions followed 6 steps using the structured SPIRIT intervention guide, which included prescribed questions based on the intervention’s theoretical underpinnings^26,27^ to (1) assess illness representations, (2) identify goals and concerns, (3) create conditions for conceptual change, (4) introduce replacement information, (5) summarize, and (6) set goals.^21^ The first session was conducted in a private room in the clinic or using video conferencing. The brief second session, which was optional, was conducted approximately 2 weeks later to address concerns raised after the first session or to include another family member if the dyad indicated that person might be involved in EOL decision-making. The champion documented the patient’s EOL preferences and the surrogate’s name, relationship to the patient, and contact information in the medical record.

Two aspects of intervention delivery were standardized. Champions had to use the intervention guide, and sessions could be conducted in person or using video conferencing (but not by telephone) to optimize exchanges between patient and surrogate and to capture nonverbal communication. After September 2020, all sessions were delivered using video conferencing to adhere to social-distancing policies. The monthly caseload for SPIRIT delivery per champion was approximately 1 dyad so that conducting sessions would not be burdensome.

Forty-four champions were certified after completing comprehensive training that included guided self-study; a full-day, competency-based workshop; evaluations of video-recorded practice sessions between champions and volunteers; and a 2.5-hour debriefing. After September 2020, all champions participated in a 1.5-hour training session focused on conducting sessions remotely using video conferencing.

To monitor intervention fidelity, the project manager at the coordinating center reviewed self-evaluation checklists completed by champions at the end of each session. The checklist documented start and finish times, steps completed, and notes regarding challenges or barriers. At the 2-week follow-up, a research assistant queried the patient and surrogate about the extent of components covered by the champion.

### Control Condition

All sites were in states endorsing physician orders for a scope of treatment paradigm. Usual ACP care involved a social worker handing written information on ADs to a patient on their first day of dialysis and addressing questions as required by the Centers for Medicare & Medicaid Services.^28^ This typically took about 10 minutes. If completed, the AD was documented on the plan of care. If a patient expressed a desire not to be resuscitated, a do-not-resuscitate order was written by a nephrologist and placed in the medical record. A social worker or charge nurse updated the code status annually.

### Outcomes

#### Primary Outcomes

As in previous SPIRIT trials,^22,23,25^ we chose 2 weeks to observe immediate effects on preparedness outcomes. Dyad congruence was assessed using the goals-of-care tool,^24^ which included 2 scenarios describing common medical conditions in patients with kidney failure. In the first scenario, the patient develops a severe complication and cannot speak for themself. The medical team believes recovery is unlikely and continuing life-sustaining treatment, including dialysis, will no longer be beneficial. In the second scenario, the patient develops advanced dementia. Each scenario had 3 response options: (1) “The goals of care should focus on delaying my death, and thus I want to continue life-sustaining treatment.” (2) “The goals of care should focus on my comfort and peace, and thus I do not want life-sustaining treatment, including dialysis.” and (3) “I am not sure.” Patients and surrogates completed this tool independently, and their responses were compared to determine congruence, either congruent in both scenarios or incongruent. If both endorsed “I am not sure,” they were considered incongruent.

Patient decisional conflict was measured using the 13-item Decisional Conflict Scale, which ranges from 1 to 5, in which higher scores indicate greater difficulty in weighing benefits and burdens of life-sustaining treatments.^29^ Surrogate decision-making confidence was measured using a 5-item Decision-Making Confidence scale, which ranges from 0 to 4, with higher scores indicating greater comfort in performing as a surrogate.^30^ As in previous SPIRIT trials,^23,24^ we created a composite outcome^31^ by combining dyad congruence and surrogate decision-making confidence to differentiate surrogates who understood the patients’ wishes and felt confident in their role from those who felt confident when they in fact did not know the patients’ wishes.

#### Secondary Outcomes

We selected 3 months for bereavement outcome assessment based on our efficacy data.^24^ Symptoms of anxiety and depression were measured using the Hospital Anxiety and Depression Scale, which ranges from 0 to 21 for both subscores, with higher scores indicating greater symptom severity.^32^ Posttraumatic distress symptoms were assessed using the Post-Traumatic Stress Symptoms Scale–10, which ranges from 10 to 70, with higher scores indicating more intense symptoms.^33^

### Statistical Analysis

The baseline characteristics between the intervention and control groups were compared using generalized estimating equation methods, accounting for within-clinic correlation. Analyses of preparedness and bereavement outcomes were intention to treat with all available data. Preparedness was analyzed using generalized estimating equations, and bereavement outcomes were analyzed using generalized linear mixed models, adjusting for baseline values and accounting for within-clinic correlation. We used the logistic link function for binary outcomes and the identity link for continuous outcomes.

To account for possible COVID-19 pandemic effects on bereavement outcomes, we created a variable to indicate the timing of baseline and 3-month assessment relative to the date of the COVID-19 emergency declaration: pre-pre declaration (both assessments completed before March 13, 2020), pre-post (baseline completed before March 13, 2020, and 3-month follow-up completed after March 13, 2020), and post-post (both completed after March 13, 2020). We examined the interaction between treatment group and assessment timing to assess the COVID-19 pandemic’s influence on the intervention effect. We did not use the assessment timing indicator for the preparedness outcomes because 73% of those outcomes were collected before the COVID-19 pandemic.

Power analysis was based on mixed-effects models, 2-sided α = .05, and intraclass correlation coefficients ranging from 0.01 to 0.04, observed in prior work from our group.^23,24^ We estimated a sample size of 400 dyads from 29 clinics to have 80% power to detect an odds ratio (OR) of 1.8 for the composite outcome. Statistical analyses were performed using R, version 4.3.1 (R Project for Statistical Computing).

## Results

### Participant Enrollment, Characteristics, and Dialysis Clinics

Of the 850 eligible patients, consent was obtained for 474 dyads (55.8%); of those, 426 dyads completed the baseline measurements (Table 1). Of the 426 dyads enrolled, 231 were in the intervention clinics, and 195 were in the control clinics. Among all dyads, the mean (SD) patient age was 61.9 (12.7) years, and the mean (SD) surrogate age was 53.7 (15.4) years.

**Table 1.** Characteristics of Patients Receiving Dialysis and Their Surrogate Decision-Makers^a^

Characteristic	Individuals, No. (%)	
	Intervention group (n = 231 dyads)	Control group (n = 195 dyads)
**Patients**		
Age, mean (SD), y	61.5 (12.8)	62.4 (12.5)
Sex		
Female	121 (52.4)	97 (49.7)
Male	110 (47.6)	98 (50.3)
Race and ethnicity^b^		
Hispanic Black	1 (0.4)	3 (1.5)
Hispanic White	4 (1.7)	2 (1.0)
Non-Hispanic Black	160 (62.3)	129 (66.2)
Non-Hispanic White	62 (26.8)	51 (26.2)
Other^c^	4 (1.7)	10 (5.1)
High school graduate or above	194 (84.0)	165 (84.6)
Traditional center hemodialysis	206 (89.2)	193 (99.0)^d^
Time treated with dialysis, median (IQR), y	2.4 (1.1-5.4)	3.5 (1.6-6.7)^e^
**Surrogate decision-makers**		
Age, mean (SD), y	53.2 (14.7)	54.3 (16.1)
Sex		
Female	168 (72.7)	152 (77.9)^f^
Male	63 (27.3)	43 (22.1)
Race and ethnicity^b^		
Hispanic Black	5 (2.2)	4 (2.1)
Hispanic White	1 (0.4)	4 (2.1)
Non-Hispanic Black	154 (66.7)	128 (65.6)
Non-Hispanic White	63 (27.3)	53 (27.2)
Not reported	1 (0.4)	1 (0.5)
Other^c^	7 (3.0)	5 (2.6)
High school graduate or above	206 (89.2)	173 (89.2)^g^
Relationship to patient		
Spouse or partner^h^	83 (35.9)	61 (31.3)
Child or son-in-law or daughter-in-law	70 (30.3)	75 (38.5)
Sibling	28 (12.1)	24 (12.3)
Friend	19 (8.2)	10 (5.1)
Parent	16 (6.9)	16 (8.2)
Other	15 (6.5)	9 (4.6)

^a^ All *P* values for comparisons between intervention and control groups were based on the generalized estimating equations method to account for within-clinic correlation.

^b^ Proportions of non-White vs White were compared.

^c^ Other included Asian, Native American, and more than 1 race and/or ethnicity.

^d^ A higher percentage of patients in the control group were receiving traditional center hemodialysis (*P* = .03).

^e^ Slightly longer mean years treated with dialysis (*P* = .002).

^f^ Higher proportion of female surrogates (*P* < .001).

^g^ One participant refused to answer.

^h^ Proportions of spouse or partner vs nonspouse or nonpartner were compared.

Among the 231 patients in the intervention group, the mean (SD) age was 61.5 (12.8) years; 121 (52.4%) were female and 110 (47.6%) were male; 1 (0.4%) was Hispanic Black, 4 (1.7%) were Hispanic White, 160 (62.3%) were non-Hispanic Black, 62 (26.8%) were non-Hispanic White, and 4 (1.7%) were categorized as other. Among the 195 patients in the control group, the mean (SD) age was 62.4 (12.5) years; 97 (49.7%) were female and 98 (50.3%) were male; 3 (1.5%) were Hispanic Black, 2 (1.0%) were Hispanic White, 129 (66.2%) were non-Hispanic Black, 51 (26.2) were non-Hispanic White, and 10 (5.1%) were categorized as other.

Among the 231 surrogates in the intervention group, the mean (SD) age was 53.2 (14.7) years; 168 (72.7%) were female and 63 (27.3%) were male; 5 (2.2%) were Hispanic Black, 1 (0.4%) was Hispanic White, 154 (66.7%) were non-Hispanic Black, 63 (27.3%) were non-Hispanic White, and 7 (3.0%) were categorized as other. Race and ethnicity was not reported for 1 surrogate (0.4%). Among the 195 surrogates in the control group, the mean (SD) age was 54.3 (16.1) years; 152 (77.9%) were female and 43 (22.1%) were male; 4 (2.1%) were Hispanic Black, 4 (2.1%) were Hispanic White, 128 (65.6%) were non-Hispanic Black, 53 (27.2%) were non-Hispanic White, and 5 (2.5%) were categorized as other. Race and ethnicity was not reported for 1 surrogate (0.5%).

Initially, 29 dialysis clinics were randomized. In August 2018, 4 additional clinics in Georgia were recruited and randomized, but 3 dialysis clinics owned by the same organization withdrew soon after randomization due to organizational problems. No participants had been recruited from those clinics. In January 2019, 1 underperforming site with 8 clinics (3 in the intervention and 5 in the control group) was replaced after enrolling 8 dyads with a new site and state with 9 clinics, resulting in 42 dialysis clinics being approached and randomized. Those 9 clinics in the new site were randomized with a 2:1 ratio (6 to the intervention group and 3 to the control group) to compensate for slower enrollment in the intervention group. The power recalculated at that time was greater than 80%. After the 3 dialysis clinics withdrew soon after randomization, of the 39 remaining clinics, 31 (79.5%) were not for profit, and 24 (61.5%) had a medium to large patient census.

After their 2-week follow-up , 167 intervention and 175 control dyads were retained for 9 months (the original end of study participation) (Figure); of those, 82 dyads (49.1%) in the intervention group and 84 (48.0%) in the control group consented to remain on study for an additional 12 months. The last participant follow-up was completed in January 2023.

**Figure.**
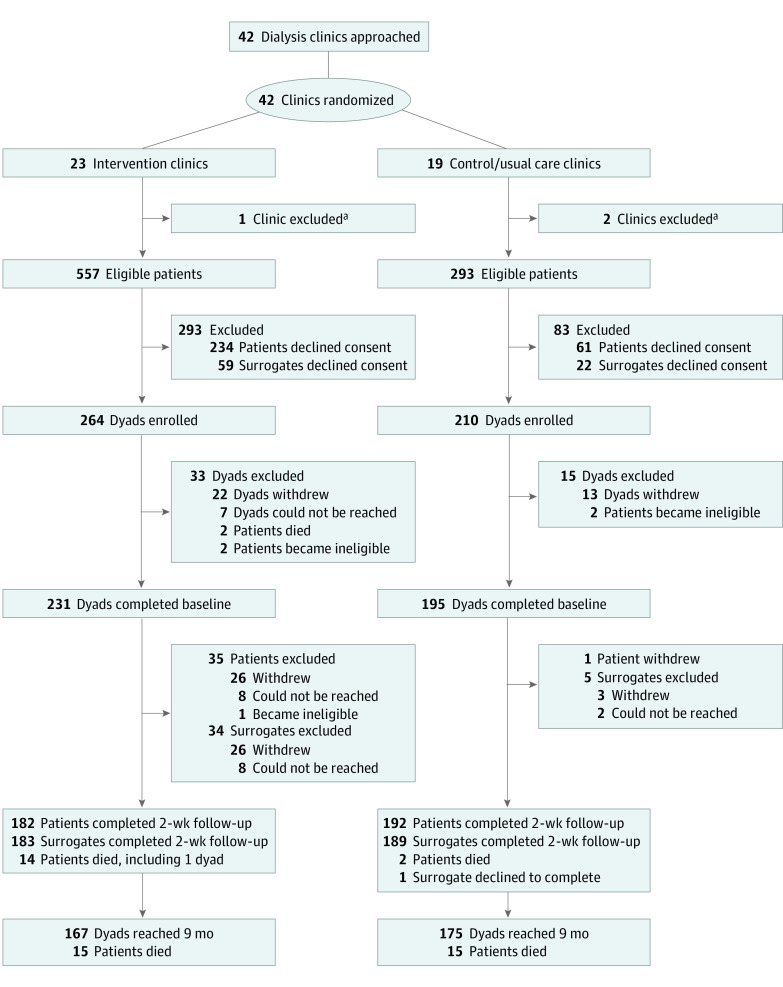
CONSORT Diagram CONSORT indicates Consolidated Standards of Reporting Trials. ^a^Three dialysis clinics owned by the same dialysis provider organization withdrew soon after randomization due to organizational problems.

### Intervention Fidelity

Of the 231 intervention dyads, 191 (82.7%) completed SPIRIT session 1, and 146 of 191 (76.4%) completed session 2. The median duration of session 1 was 60 minutes (range, 30-150 minutes), and the median duration of session 2 was 15 minutes (range, 5-75 minutes). Most sessions were conducted in person in the clinics (session 1: 162 of 191 [84.8%]; session 2: 85 of 146 [58.2%]). The champion checklists indicated that each of the 6 intervention steps was completed in 98% to 100% of sessions. Patients (n = 182) reported that the topic least frequently explored by the champion was having made tough medical decisions in the past (“definitely no”; n = 27 [14.8%]). Surrogates (n = 183) reported that the least frequently explored topic was potential family conflict (“definitely no”; n = 18 [9.8%]).

### Preparedness Outcomes at 2 Weeks

Compared with control dyads, adjusting for baseline values, intervention dyads were more likely to be congruent on goals of care (OR, 1.61; 95% CI, 1.12-2.31; *P* = .001) (Table 2) and had lower decisional conflict (β, −0.10; 95% CI, −0.13 to −0.07; *P* < .001). There was no statistically significant difference in decision-making confidence scores between groups (β, 0.06; 95% CI, −0.01 to 0.13; *P* = .12), but the intervention dyads were more likely to have a positive composite outcome compared with controls (OR, 1.57; 95% CI, 1.06-2.34; *P* = .03).

**Table 2.** Preparedness Outcomes of Sharing Patients’ Illness Representations to Increase Trust Intervention for Patients Receiving Dialysis and Their Surrogates^a^

Outcome	Individuals, No. (%) or mean (SD)		OR or β (95% CI)	*P* value
	Intervention group	Control group		
Congruent dyads^b^				
Baseline (n = 231 [in intervention group], 195 [in control group])	98 (42.4)	76 (39.0)	1.61 (1.12 to 2.31)	.001
2 wk (n = 182, 187)	111 (61.0)	94 (50.3)		
Patient DCS score^c^				
Baseline (n = 231, 194)^d^	2 (0.5)	1.9 (0.4)	−0.10 (−0.13 to −0.07)	<.001
2 wk (n = 182, 192)	1.8 (0.5)	1.9 (0.4)		
Surrogate DMC score^e^				
Baseline (n = 231, 195)	3.6 (0.5)	3.7 (0.5)	0.06 (−0.01 to 0.13)	.12
2 wk (n = 183, 189)	3.8 (0.3)	3.7 (0.4)		
Composite outcome^f^				
Baseline (n = 231, 195)	92 (39.8)	74 (38.0)	1.57 (1.06 to 2.34)	.03
2 wk (n = 182, 187)	107 (58.8)	90 (48.1)		

Abbreviations: DCS, Decisional Conflict Scale; DMC, Decision-Making Confidence; OR, odds ratio.

^a^ Intervention effect on 2-week scores was obtained using the generalized estimating equations, adjusted for baseline values and accounting for within-clinic correlation. Participants with 2-week values were included in the model. Data are reported as number (percentage) and OR (95% CI) for congruent dyads and composite outcome or as mean (SD) and β (95% CI) for patient DCS and surrogate DMC.

^b^ Congruent dyads in both scenarios of the goals-of-care document, except that both patient and surrogate chose “not sure.”

^c^ Scores range from 1 to 5, with higher scores indicating greater conflict and difficulty.

^d^ One patient did not complete all items of the scale.

^e^ Scores range from 0 to 4, with higher scores indicating greater confidence.

^f^ Grouped into either dyads congruent in both scenarios and a surrogate DMC score of 3 or greater or not; data indicate dyads congruent in both scenarios and a surrogate DMC score of 3 or greater.

### Bereavement Outcomes at 3 Months After Patient Death

By the 9-month follow-up, 29 intervention patients (12.5%) and 17 control patients (8.7%) had died. During the extended follow-up, 43 additional patients died, yielding a total of 89 deaths (54 patients in the intervention group and 35 in the control group). Survival time was similar between groups (hazard ratio, 1.25; 95% CI, 0.78-1.99; *P* = .35). Of the 89 surrogates, 77 (86.5%), which included 46 in the intervention group and 31 in the control group, completed bereavement outcomes. Their mean (SD) age was 55.6 (14.4) years, 65 (84.4%) were women, 3 (3.9%) were Hispanic White, 50 (64.9%) were non-Hispanic Black, and 24 (31.2%) were non-Hispanic White. These characteristics were similar between groups.

Adjusting for baseline values and assessment timing, intervention surrogates had lower anxiety scores than did controls (β, −1.55; 95% CI, −3.08 to −0.01; *P* = .05), but there were no group differences in depression (β, −0.18; 95% CI, −2.09 to 1.73; *P* = .84) or posttraumatic distress (β, −0.96; 95% CI, −7.39 to 5.46; *P* = .75) (Table 3). Table 4 presents bereavement outcomes by assessment timing relative to the COVID-19 emergency declaration. For all bereavement outcomes, there was no interaction between treatment group and assessment timing. In both groups, those with pre-post assessment timing had significantly higher anxiety (β, 1.87; 95% CI, 0.27-3.46; *P* = .02), depression (β, 1.90; 95% CI, 0.55-3.24; *P* = .007), and posttraumatic distress (β, 6.40; 95% CI, 1.31-11.49; *P* = .01) than did surrogates with pre-pre assessment timing.

**Table 3.** Bereavement Outcomes of Sharing Patients’ Illness Representations to Increase Trust Intervention for Surrogates^a^

Outcome	Individuals, mean (SD)		β (95% CI)	*P* value
	Intervention group	Control group		
HADS anxiety^b^				
Baseline	4.8 (3.0)	4.9 (2.8)	−1.55 (−3.08 to −0.01)	.05
3 mo	5.6 (3.6)	6.7 (3.7)		
HADS depression^b^				
Baseline	3.3 (2.6)	2.8 (2.8)	−0.18 (−2.09 to 1.73)	.84
3 mo	4.3 (2.9)	4.2 (2.7)		
PTSS-10^c^				
Baseline	20.0 (9.2)	22.0 (9.9)	−0.96 (−7.39 to 5.46)	.75
3 mo	26.4 (12.2)	29.3 (13.3)		

Abbreviations: HADS, Hospital Anxiety and Depression Scale; PTSS-10, Post-Traumatic Stress Symptoms Scale–10.

^a^ Obtained using linear mixed models, adjusted for baseline values and assessment timing relative to the COVID-19 emergency declaration (March 13, 2020), and with a random intercept for clinic. Participants with 3-month values were included in the model. Sample sizes for the intervention group are 54 at baseline and 46 at 3 months and 35 at baseline and 31 at 3 months for the control group.

^b^ Scores range from 0 to 21, with higher scores indicating greater symptom severity.

^c^ Scores range from 10 to 70, with higher scores indicating greater symptom severity.

**Table 4.** Bereavement Outcomes of Sharing Patients’ Illness Representations to Increase Trust Intervention for Surrogates by Assessment Timing Relative to the COVID-19 Emergency Declaration and by Group

Outcome	Individuals, mean (SD)					
	Pre-pre^a^		Pre-post^b^		Post-post^c^	
	Intervention (n = 15)	Control (n = 15)	Intervention (n = 27)	Control (n = 13)	Intervention (n = 4)	Control (n = 3)
HADS anxiety^d^						
Baseline	6.0 (3.5)	3.9 (2.5)	4.3 (2.7)	5.2 (3.2)	4.5 (3.1)	5.7 (2.5)
3 mo	5.1 (3.2)	6.0 (4.1)	6.4 (3.5)	8.3 (2.8)	2.3 (3.9)	3.3 (2.5)
HADS depression^d^						
Baseline	4.2 (1.9)	3.0 (3.0)	2.6 (2.4)	2.7 (2.7)	1.8 (2.2)	1.3 (2.3)
3 mo	3.8 (3.1)	3.5 (2.6)	4.8 (2.8)	5.4 (2.2)	2.5 (3.1)	2.7 (3.8)
PTSS-10^e^						
Baseline	21.5 (9.3)	19.1 (6.4)	18 (5.9)	24.2 (10.9)	19.8 (13.0)	26.7 (21.1)
3 mo	23.4 (9.6)	25.5 (12.7)	28.4 (11.6)	35.1 (11.9)	23.5 (23.0)	23 (17.3)

Abbreviations: HADS, Hospital Anxiety and Depression Scale; PTSS-10, Post-Traumatic Stress Symptoms Scale–10.

^a^ Both baseline and 3-month follow-up were completed before March 13, 2020.

^b^ Baseline was completed before March 13, 2020, and 3-month follow-up was completed after March 13, 2020.

^c^ Both baseline and 3-month follow-up were completed after March 13, 2020.

^d^ Scores range from 0 to 21, with higher scores indicating greater symptom severity.

^e^ Scores range from 10 to 70, with higher scores indicating greater symptom severity.

## Discussion

In this cluster randomized clinical trial (An Effectiveness-Implementation Trial of SPIRIT in ESRD) conducted in dialysis clinics in 5 US states using broad inclusion criteria and minimal control over intervention delivery, SPIRIT was superior to a usual care control group with better 2-week outcomes in dyad congruence on goals of care, patient decisional conflict, and the composite outcome combining dyad congruence and surrogate decision-making confidence. The SPIRIT intervention was superior to usual care control with lower surrogate anxiety at 3-month bereavement but did not influence depression or posttraumatic distress. These intervention effects on preparedness outcomes are consistent with previous SPIRIT trials.^22,23,24,25^ In contrast, the current bereavement findings differ from those observed in the efficacy trial in which compared with control, SPIRIT showed significantly lower anxiety, depression, and posttraumatic distress.^24^ As higher bereavement distress was seen among surrogates with pre-post assessment timing, the bereavement outcomes may have been affected by COVID-19 pandemic-related distress.^34,35,36,37^ In contrast, the preparedness outcomes may not have been as affected by the COVID-19 pandemic because 73% of these were collected before the pandemic.

The 10% reduction in decisional conflict in the intervention group when there was no such reduction in the control group supports that SPIRIT delivered by health care workers at dialysis centers can help patients engage in ACP discussions. The changes in surrogate anxiety observed in the pre-pre COVID-19 pandemic subgroup (a 15% decrease in the intervention group and a 54% increase in the control group) reflect a meaningful intervention effect.

Trials^14,15,38,39^ aimed at increased AD documentation, goal-concordant care, and reduced health care use have largely failed to demonstrate treatment effects on those outcomes, likely due to pitfalls associated with ADs^13^ or low intervention fidelity.^14^ Unlike trials that focused on such system-level outcomes, we focused on patient-centered and family-centered outcomes consistent with the level and goal of intervention. The SPIRIT intervention was designed to provide both patients and surrogates an opportunity to mentally rehearse complex EOL decision-making and implications for the patient and family if choosing aggressive care vs comfort-oriented care. The SPIRIT intervention is a testable model of ACP discussions as opposed to idiosyncratic ACP conversations that may occur in practice and are difficult to replicate. The benefits of SPIRIT on patient and surrogate preparedness for EOL decision-making and surrogate bereavement outcomes should not be taken lightly. It has been well documented that family members of patients receiving dialysis lack an understanding of patients’ EOL preferences,^5^ EOL decision-making is too complex to be addressed by AD alone,^40^ and consequences of a lack of preparedness can be profound.^41,42,43,44,45^ The present data support findings that jointly preparing patients and surrogates for EOL using SPIRIT has been shown to be beneficial.

The high intervention fidelity in this trial is noteworthy. The champions delivered SPIRIT sessions with a high degree of adherence to intervention components, perhaps due to the well-tested, competency-based training that included clear instructions about the rationale and intent of each question. Champions were trained to follow the SPIRIT intervention guide at all times because previous SPIRIT trials^22,23,24^ revealed that following the guide could move the ACP discussion smoothly forward and could help to ensure the quality and consistency of intervention delivery within and across interventionists. Another factor in the high champion performance was that the monthly caseload for SPIRIT delivery was decided jointly by the champions and the study team.

### Limitations

This study has limitations. We chose a clinic-level cluster design to control for geographic location, health care system, and clinic-specific characteristics (eg, state-level or clinic-level ACP initiatives). Although this is a frequently used design for pragmatic trials, it is not risk free.^46^ The clinics could not be blinded to treatment allocation, which may explain the high refusals in intervention clinics in which their patients would have been certain of being assigned to SPIRIT. However, we minimized potential bias by blinding outcome assessors and by prespecifying primary outcomes, assessment times, and analyses. Unlike pragmatic trials that rely on electronic health record data, we used patient-reported and surrogate-reported outcomes because, while limiting generalizability, they provide a more comprehensive assessment of preparedness for EOL decision-making.

## Conclusions

The findings of this pragmatic clinic-level randomized trial of an ACP intervention, SPIRIT, in dialysis clinics across 5 states with a diverse sample of patients and their surrogates demonstrated its effectiveness on patient and surrogate preparedness for EOL decision-making. The effectiveness of SPIRIT on bereavement outcomes for surrogates was mixed and potentially influenced by psychosocial effects of the COVID-19 pandemic. The ACP intervention implemented in dialysis centers may be an effective strategy to the dyad preparation for EOL care as opposed to the current focus on ADs.

## Notes

Supplement 1.
Trial Protocol

Supplement 2.
Data Sharing Statement
