# Supplementary material for: Effectiveness of an Advance Care Planning Intervention in Adults Receiving Dialysis and Their Families: A Cluster Randomized Clinical Trial
Source: JAMA Netw Open. 2024 Jan 30;7(1):e2351511. doi: 10.1001/jamanetworkopen.2023.51511 (PMC10828909; doi:10.1001/jamanetworkopen.2023.51511)
Supplement: Supplement 1. — Trial Protocol [file jamanetwopen-e2351511-s001.pdf]

An Effectiveness-Implementation Trial of SPIRIT in ESRD (Short title: SPIRIT Trial)

National Clinical Trial (NCT) Identified Number: NCT031385464

Principal Investigator: Mi-Kyung Song

Funded by: NIH/NINR, R01NR017018

Version Number: v.13

8 April 2021

Table of Contents

STATEMENT OF COMPLIANCE 1
1 PROTOCOL SUMMARY 2
1.1 Synopsis 2
1.2 Schedule of Activities (SoA): Randomization occurs at the clinic level 4
2 INTRODUCTION 4
2.1 Study Rationale 5
2.2 Background 5
2.3 Risk/Benefit Assessment 9
2.3.1 Known Potential Risks 9
2.3.2 Known Potential Benefits 9
2.3.3 Assessment of Potential Risks and Benefits 10
3 OBJECTIVES AND ENDPOINTS 10
4 STUDY DESIGN 12
4.1 Overall Design 12
4.2 Scientific Rationale for Study Design 12
4.3 Justification for INTERVENTION 13
4.4 End of Study Definition 13
5 STUDY POPULATION 13
5.1 Inclusion Criteria 13
5.2 Exclusion Criteria 13
5.3 Lifestyle Considerations 14
5.4 Screen Failures 14
5.5 Strategies for Recruitment and Retention 14
6 STUDY INTERVENTION(s) 15
6.1 Study Intervention(s) Administration 15
6.1.1 Study Intervention Description 15
6.1.2 Dosing and Administration 16
6.2 Preparation/Handling/Storage/Accountability 16
6.2.1 INTERVENTIONIST TRAINING and accountability 16
6.2.2 Formulation, Appearance, Packaging, and Labeling 17
6.2.3 Product Storage and Stability 17
6.2.4 Preparation 17
6.3 Measures to Minimize Bias: Randomization and Blinding 17
6.4 Study Intervention Compliance 17
6.5 Concomitant Therapy 18
6.5.1 Rescue Medicine 18
7 STUDY INTERVENTION DISCONTINUATION AND PARTICIPANT DISCONTINUATION/WITHDRAWAL 18
7.1 Discontinuation of Study Intervention 18
7.2 Participant Discontinuation/Withdrawal from the Study 18
7.3 Lost to Follow-Up 18
8 STUDY ASSESSMENTS AND PROCEDURES 19
8.1 OUTCOME Assessments 19
8.2 Safety and Other Assessments 21
8.3 Adverse Events and Serious Adverse Events 21
8.3.1 Definition of Adverse Events (AE) 21
8.3.2 Definition of Serious Adverse Events (SAE) 22

8.3.3 Classification of an Adverse Event 22
8.3.4 Time Period and Frequency for Event Assessment and Follow-Up 22
8.3.5 Adverse Event Reporting 22
8.3.6 Serious Adverse Event Reporting 22
8.3.7 Reporting Events to Participants 23
8.3.8 Events of Special Interest 23
8.3.9 Reporting of Pregnancy 23
8.4 Subject safety 23
8.4.1 Definition of Unanticipated Problems (UP) 23
8.4.2 Unanticipated Problem Reporting 23
8.4.3 Reporting Unanticipated Problems to Participants 23
9 STATISTICAL CONSIDERATIONS 23
9.1 Statistical Hypotheses 24
9.2 Sample Size Determination 24
9.3 Populations for Analyses 25
9.4 Statistical Analyses 25
9.4.1 General Approach 25
9.4.2 Analysis of the Primary Endpoint(s) 25
9.4.3 Analysis of the Secondary Endpoint(s) 25
9.4.4 Safety Analyses 26
9.4.5 Baseline Descriptive Statistics 26
9.4.6 Planned Interim Analyses 26
9.4.7 Sub-Group Analyses 26
9.4.8 Tabulation of Individual participant Data 26
9.4.9 Exploratory Analyses 26
10 SUPPORTING DOCUMENTATION AND OPERATIONAL CONSIDERATIONS 26
10.1 Regulatory, Ethical, and Study Oversight Considerations 26
10.1.1 Informed Consent Process 26
10.1.2 Study Discontinuation and Closure 28
10.1.3 Confidentiality and Privacy 28
10.1.4 Future Use of Stored Specimens and Data 28
10.1.5 Key Roles and Study Governance 29
10.1.6 Safety Oversight 30
10.1.7 Clinical Monitoring 31
10.1.8 Quality Assurance and Quality Control 31
10.1.9 Data Handling and Record Keeping 32
10.1.10 Protocol Deviations 33
10.1.11 Publication and Data Sharing Policy 33
10.1.12 Conflict of Interest Policy 34
11 REFERENCES 34

STATEMENT OF COMPLIANCE

The trial will be carried out in accordance with International Conference on Harmonisation Good Clinical Practice (ICH GCP) and the following:

- United States (US) Code of Federal Regulations (CFR) applicable to clinical studies (45 CFR Part 46, 21 CFR Part 50, 21 CFR Part 56, 21 CFR Part 312, and/or 21 CFR Part 812)

  National Institutes of Health (NIH)-funded investigators and clinical trial site staff who are responsible for the conduct, management, or oversight of NIH-funded clinical trials have completed Human Subjects Protection and ICH GCP Training.

  The protocol, informed consent form(s), recruitment materials, and all participant materials have been approved by the Institutional Review Board (IRB) at:

  Emory (Study No.: IRB0094859; approved on 3/3/2017)
  University of New Mexico (Study No: 17-357 approved on 10-27.17)
  University of Pittsburgh (Study No.: PRO07070106; approved on 09/08/17)
  University of North Carolina at Chapel Hill (Study #: 17-1295; approved on 7/28/2017)

  RRI Carolina Dialysis approval (08/03/2017)
  FMC approval 4.1.18
  DCI approval & IRBAA 1.22.18

  Any amendment to the protocol requires review and approval by the IRB before the changes are implemented to the study. In addition, all changes to the consent form will be IRB-approved.

1 PROTOCOL SUMMARY

1.1 SYNOPSIS

**Title:**	An Effectiveness-Implementation Trial of SPIRIT in ESRD
**Study Description:**	This multicenter, clinic-level cluster randomized trial will evaluate the effectiveness of SPIRIT, an advance care planning (ACP) intervention, delivered by dialysis care providers as part of routine care in free-standing outpatient dialysis clinics compared to usual care. Simultaneously, we will evaluate the implementation of SPIRIT, including its sustainability.
**Objectives:**	Primary Aim 1. Examine the effectiveness of SPIRIT compared to usual care on preparedness outcomes for end-of-life decision making (defined as dyad congruence on goals of care, patient decisional conflict, and surrogate decision-making confidence) at 2 weeks post-intervention<br>Aim 2. Evaluate the process outcomes of SPIRIT implementation: acceptability, fidelity, intervention costs, and sustainability during the initial and delayed implementation of SPIRIT (Descriptive aim to generate data for translation)<br>Aim 3. Examine the effectiveness of SPIRIT and usual care on surrogates' post-bereavement distress (anxiety, depression, and post-traumatic distress symptoms) at 3 months after the patient's death<br>Aim 4 (exploratory): Examine the effectiveness of SPIRIT and usual care on end-of-life treatment intensity (healthcare utilization; percentages of patients hospitalized, having ICU admission, and having intensive procedures and length of hospital stay) during the final month of life
**Endpoints:**	Primary Endpoints (Aim 1):<br>Dyad congruence on goals of care, patient decisional conflict, and surrogate decision-making confidence at 2 week post-intervention<br>Secondary Endpoints (Aim 3):<br>Surrogate HADS and PTSS scores at 3 months after the patient's death;<br>Exploratory (Aim 4):<br>EOL treatment intensity
**Study Population:**	400 patients and 400 surrogates of the patients participate as pairs (400 patient-surrogate dyads); adults (>=18 y); both genders; all race and ethnicity; ESRD/chronic dialysis population
**Phase:**	**Phase III effectiveness trial**
**Description of Sites/Facilities Enrolling Participants:**	Participants recruited from outpatient dialysis centers located in GA (Emory), NC (UNC-CH), VA (UVA) and PA (U of Pitt).; dialysis centers owned and managed by Emory Healthcare, Fresenius Medical Care (FMC), Renal Research Institute (RRI), and Dialysis Clinic Inc (DCI). A total of 29 free-standing dialysis centers (initial).
**Description of Study Intervention:**	**SPIRIT** (**S**haring **P**atient's **I**llness **R**epresentation to **I**ncrease **T**rust), a patient and family-centered ACP intervention based on the Representational Approach to Patient Education, is to establish a testable model of how end-of-life care discussions could occur between a dialysis patient and his/her chosen surrogate (usually a spouse or adult child). The discussions, which are facilitated by a trained care provider, are framed around addressing each individual's representations of (beliefs about) the illness and views of life-sustaining measures at the end of life. SPIRIT follows a six-step learning objective over two-sessions, which together take about 60 minutes.
**Study Duration:**	60 months

Participant Duration: For patients, baseline and 2-week follow up (active participation), and then an observational period for 9 months (or until death). We will request extension of 12 additional months at completion of 9 month time point.

For surrogates, baseline and 2-week follow-up (active participation), and then a 9 month observational period. We will request extension of 12 additional months if patient is still living. A post-death follow-up survey at 3 months after the patient death (if the patient death occurs during the observational period).

1.2 SCHEDULE OF ACTIVITIES (SOA): Randomization occurs at the clinic level

**Days offset**	**Procedure**	**Note**
Patients and surrogates	Patient referrals[a]	Screened and willing patients
	Patient screened (by research staff) [b]	Linked to eligibility checklist
Day -14 to Day -7	Obtaining patient informed consent in person[b]	Scan and upload signed consent pdf
	Obtaining contact information[b]	
	Medical record review: clinical characteristics[b]	To be linked to "pt consent"
	Usual care review 1[b]	To be linked to "pt consent" & separate<br>Every 6 months
Day 0	Surrogate verbal consent[b]	
	Surrogate verbal consent received[b]	
	Scheduling baseline (T1) appointment	If control, schedule both T1 & T2
	Reminder call for T1	
Day 14 (+/- 7)	**Baseline (T1)-patient completion**	Link to the measures
	**Baseline (T1)-surrogate completion**	Link to the measures
	Payment (T1) mailed to patient	With thank-you note
	Payment (T1) mailed to surrogate	With thank-you note
	Scheduling SPIRIT Session I & II and 2-wk F/U[b]	Need to coordinate with the care provider for SPIRIT sessions
	**SPIRIT Session I[a]**	
	SPIRIT Session II reminder call[b]	Two days prior
	**SPIRIT Session II[a]**	
	SPIRIT Interview Guide Checklist[b]	Care provider interventionist completes and the Site Coordinator enters to REDCap
	Reminder call for T2	Central
	**2-week follow-up (T2)-patient completion**	2 weeks from SPIRIT; including acceptability survey and SPIRIT components
	**2-week follow-up (T2)-surrogate completion**	2 weeks from SPIRIT; including acceptability survey and SPIRIT components
	Payment (T2) mailed to patient	With thank-you note
	Payment (T2) mailed to surrogate	With thank-you note
	Monthly check-in call 1	1 month from T2
	Monthly check-in call 2	
	Monthly check-in call 3	
	Monthly check-in call 4	
	Monthly check-in call 5	
	Usual care review 2[a]	
	Monthly check-in call 6	
	Monthly check-in call 7	
	Monthly check-in call 8	
	Monthly check-in call 9	With note on study completion
	Mailing sympathy card	Upon patient death
	Contacting surrogate & scheduling a 3-month post-death F/U	
	Postcard reminder 2 weeks before the F/U	
	Reminder call for 3-month F/U	
	**F/U 3 month post death**	
	Payment (F/U 3M) mailed to surrogate	With thank-you note
	Clinic-level contextual data[b]	At the end of each implementation
Care providers	Informed consent[b]	At the end of each implementation
	**Acceptability assessment[b]**	Brief survey and interview
	**SPIRIT care provider interview-sustainability[b]**	
[a] Performed locally by the chosen care provider; [b] Procedures performed locally by research personnel		

2 INTRODUCTION

2.1 STUDY RATIONALE

End-stage renal disease (ESRD) currently affects nearly 662,000 people in the U.S.[1] While dialysis is the treatment of choice for over 90% of patients with ESRD and is universally covered by Medicare regardless of patient age or means, the likelihood that dialysis can restore health or prolong life is limited; only 50% of dialysis patients are alive 3 years after the onset of ESRD.[1] Thus many dialysis patients and their family members or surrogate decision-makers have to face difficult end-of-life decisions. Although advance care planning (ACP), in which patients and surrogate decision-makers discuss future health states and treatment options, is a central tenet of dialysis care,[2-5] the vast majority of dialysis patients (>90%) report never engaging in ACP discussions with their care providers.[6,7] The lack of effective ACP to prepare patients and their surrogates for end-of-life decision making with sufficient time before death has deleterious consequences at all levels of society. Consequences have been well documented: prolonged use of futile treatment at the end of life, which misuses the healthcare system, high levels of surrogate distress during decision making, which emanates from not having a clear understanding of the patient's wishes, and surrogates experiencing later sequelae of psychosocial morbidities, such as depression and family discord.[8-14]

SPIRIT (**S**haring **P**atient's **I**llness **R**epresentation to **I**ncrease **T**rust), a patient and family-centered ACP intervention based on the Representational Approach to Patient Education,[15,16] was designed by our team to establish a testable model of how end-of-life care discussions could occur between a dialysis patient and his/her chosen surrogate (usually a spouse or adult child). The discussions, which are facilitated by a trained care provider, are framed around addressing each individual's representations of (beliefs about) the illness and views of life-sustaining measures at the end of life. SPIRIT follows a six-step learning objective over two-sessions, which together take about 60 minutes. The care provider, who is value-neutral, guides the patient in examining his/her values related to end-of-life care, helps the surrogate understand the patient's illness progression, and prepares the surrogate for his/her role as a surrogate in a highly emotionally charged medical setting. Over the last decade, we have iteratively tested SPIRIT to establish feasibility, patient-surrogate acceptability, and efficacy.[17-20] In these explanatory trials carried out in dialysis clinics, SPIRIT was delivered by trained research nurses. Patients and surrogates in SPIRIT showed significant improvement in preparedness for end-of-life decision making, including the extent to which: a) the patient and surrogate agreed on end-of-life care goals, b) the patient had reduced conflict about the benefits and burdens of life-sustaining treatments, and c) the surrogate had increased confidence about the role of surrogate. Key to establishing the utility of this approach for broader generalizability, surrogates who received SPIRIT reported significantly improved post-bereavement psychological outcomes after the patient's death compared to those who did not. The logical, critical next step is to ask: Will SPIRIT be effective as part of routine care in real-world clinical settings with less control? To address this very issue, we will conduct a real-world effectiveness-implementation study, an essential step prior to widespread implementation of SPIRIT.

2.2 BACKGROUND

Dialysis patients experience high end-of-life treatment intensity that may not reflect their wishes. Despite advances in dialysis, adjusted all-cause mortality rates are 6-8 times greater for dialysis patients than for individuals in the general age-matched Medicare population.[1] Only 50% of dialysis patients are alive 3 years after the onset of ESRD.[1] End-of-life treatment intensity in this population is also substantially greater than that reported for other Medicare beneficiaries with life-limiting illnesses.[21] For instance, in a study of older Medicare beneficiaries (≥ 65 years; N≅100,000) those on dialysis experienced significantly higher rates of hospitalization (76% vs 61% in cancer), ICU admission (48.9% vs 29% in cancer), and use of intensive procedures (29% vs 9% in cancer) during the final month of life.[22]

Dialysis patients and their surrogates are not knowledgeable about the natural course of ESRD. Although withdrawal of dialysis precedes 1 in 4 deaths of patients with ESRD,[23] withdrawal from dialysis and aggressive treatment is very rarely (< 6%) discussed by patients and their surrogates with sufficient time to consider alternatives such as hospice or dying at home.[23-26] In a study of over 530 chronic dialysis patients, only 19% indicated that they would want to continue dialysis if severely cognitively or functionally impaired, and 65% stated that they prefer to die at home or in hospice rather than in a hospital.[27] Patients' preferences for end of life care are significantly influenced by the patients' illness representations (e.g., perceived illness severity).[28] That is, when patients understand their illness severity and progression, they are likely to forgo futile end-of-life treatment, such as mechanical ventilation and CPR. In our work we have discovered that because so many dialysis patients are unaware that they are likely to die from an acute illness or complication (e.g., stroke, infection) rather than die from ESRD per se, they endorse the notion that with dialysis they can live as long as those without ESRD.[18,29] Similarly, surrogates are equally unaware of patients' likely illness progression and comorbidities; surrogates report that prior to the time of end-of-life decision making they have not been told that the patient's illness cannot be cured.[9] Studies indicate that spouses' judgments about dialysis patients' preferences for continuation of dialysis were only modesty correlated with patients' preferences (r = .33),[30,31] and spouses do not have intimate knowledge of whether or how long the patient would want to pursue life-sustaining measures.[32,33] Our previous work demonstrated that surrogates are overly confident about their ability to act as a surrogate in that they have little understanding of the patient's preferences and yet report confidence in understanding the patient's wishes and their role as a surrogate.[34]

Lack of preparedness for end-of-life decision-making can have a detrimental effect on patients and surrogates. There are predictable psychological and emotional consequences of lack of preparedness for end-of-life decision making. These include: high levels of conflict brought on by having to make life or death decisions (e.g., whether to withhold or withdraw mechanical ventilation, prolonging use of life sustaining measures that are deemed futile); regrets over missed opportunities to benefit from palliative care or hospice; excessive distress for family members during decision making due to interfamily conflict, time pressure to make important decisions, lack of knowledge about options; and well documented psychosocial sequelae for family members (e.g., depression, anxiety, post-traumatic stress disorder) and complicated bereavement after the patient's death.[8,13,35-43] Numerous studies, including ours, indicate that families experience greater difficulty in decision making when they are uncertain about the patient's wishes, when they feel unprepared for their role because they have never discussed it, and when they are called on to make decisions in a short period of time.[29,37,43-48] Evidence shows that this distress contributes to a high prevalence of psychiatric illness among family decision makers: in one study nearly 40% of bereaved families who experienced a loved one's death in the ICU 3 to 12 months previously had at least one psychiatric illness meeting DSM-IV criteria, such as anxiety disorder or major depression.[42] Even at 6-12 months after the patient's death, family members may experience intrusive thoughts of regret, guilt or search for evidence that they made the right decision.[8,43,45,49,50] We will test the hypothesis that surrogates' post-bereavement psychosocial distress will be reduced by SPIRIT, a patient and family-centered ACP, in a real-world setting.

Usual care does not prepare patients and surrogates for end-of-life decision making. Usual ACP in free-standing dialysis facilities is based on the Centers for Medicare & Medicaid (CMS) requirements[51] that written information on advance directives (ADs) is provided on a patient's first day of dialysis, and a member of the dialysis team (e.g., social worker) reviews the written information with patients and encourages them to complete an AD. If completed, the AD is documented on the Plan of Care forms. If a patient expresses a desire not to be resuscitated in the dialysis unit, a do-not resuscitate (DNR) order is written by a nephrologist and placed in the clinic record. If there is no DNR order in the record, a full code is presumed. CMS has recently issued two new billing codes (CPT 99497 and 99498) effective in 2016 that can be used to report the first 30 minutes and each additional 30 minutes of face-to-face services between a physician or other qualified healthcare professional and a patient, family member and/or surrogate in discussing ADs, with or without completing relevant legal forms.[52] Although this is encouraging, CMS provides neither ACP tools nor guidance to ensure consistency and quality in ACP services. CMS

recommends that ACP discussions be conducted during annual wellness visits at a doctor's office to which these codes can be added; ACP discussions occurring outside of these visits are subject to patient co-pay or deductibles, which limits utility of the policy in other care settings. Further, ACP in these codes is narrowly defined as "including the explanation and discussion of ADs" and thus it may reinforce a common pitfall in ACP, i.e., a sole focus on AD completion.[53]

SPIRIT, a patient and family-centered ACP, has beneficial effects on a range of psychosocial outcomes for dialysis patients and their surrogates. The goal of SPIRIT is to promote cognitive and emotional preparation for end-of-life decision making for patients with ESRD and their surrogates. SPIRIT is based on the Representational Approach to Patient Education.[15,16] This approach melds two theories: Leventhal's common sense model[54] and the conceptual change model.[55] The common sense model proposes that individuals have representations of their illness or health problems. Because representations are based on an individual's everyday experiences, traditional or cultural information, or media, they may not be medically accurate. However, it is critical to understand the representations because they serve as the cognitive framework that affects whether or not individuals accept or reject new information,[15] and whether knowledge is translated into behavior change.[56,57] The conceptual change model proposes that the likelihood of learning increases when the individual has an opportunity to reflect and comment on current ideas and their consequences, when the individual is dissatisfied with current ideas or recognizes their limitations, and when alternative information is seen as beneficial.[15,16,55,58] Learning/change can occur through integrating new information into existing representations to fill in gaps in understanding, through clarifying existing representations to reduce confusion, or through exchanging existing representations with new information.[58,59] The Representational Approach to Patient Education requires a care provider to elicit the patient's pre-existing illness representations before providing new information.[15,16] Then, both the care provider and the individual have an opportunity to recognize gaps or confusions in the patient's representations, and the care provider can provide new information that is specific and relevant to the person. Thus, the patient is likely to understand and act on the new information.

SPIRIT is a two-session, 60-minute, structured psychoeducational intervention, targeting both patient and surrogate. Using a provider manual, the care provider follows six steps: 1) assessing illness presentation, 2) identifying gaps and concerns, 3) creating conditions for conceptual change, 4) introducing replacement information, 5) summarizing, and 6) setting goals and planning.[60] SPIRIT first establishes an understanding of the cognitive, emotional and spiritual aspects of the patient's representation of his/her illness. This understanding enables the care provider to provide individualized medical information and to assist the patient in examining his/her own values related to life-sustaining treatment at the end of life. In this way, the patient can more readily express his/her treatment preferences to the surrogate. SPIRIT also enables the surrogate to understand the patient's illness experiences and values and to be prepared for the responsibility and emotional turmoil that can arise during decision making at the end of life. Each element of SPIRIT is designed to enhance the quality and authenticity of exchanges between patient and surrogate about experiences surrounding illness and values. During the process, the patient discovers his/her own representations about illness and dialysis and examines thresholds and/or conditions for (dis)continuing life support measures. The surrogate gains an understanding of the patient's illness experience and begins to see his/her limited life expectancy. The surrogate also validates similarities or differences with the patient in regard to life support measures and examines his/her own ability to follow the patient's wishes. This process is critical to preparation for end-of-life decision making.[20,60] To deliver SPIRIT sessions, care providers are trained in communication skills and end-of-life planning. Over 12 years of conducting iterative trials of SPIRIT with diverse dialysis patient populations, we have developed a structured protocol to assist providers in addressing the unique challenges and complexities in ACP with dialysis patients and surrogates. This evidence-based guide promotes quality, consistency, and fidelity in ACP delivery[60] in this pragmatic trial of SPIRIT.

Preliminary data to support feasibility of the proposed cluster randomized trial in dialysis clinics.
Three RCTs testing SPIRIT[18-20] were conducted in free-standing outpatient dialysis settings and established the feasibility, patient and surrogate acceptability, preliminary effects (R21NR009662), and efficacy of SPIRIT

delivered in those settings by trained research nurses. Dialysis facilities varied in type, including profit and non-profit ownership, urban and rural, and academic affiliation and community. The PI (Dr. Song) has the intimate knowledge of implementing SPIRIT in these complex health care systems. Recruitment rates have consistently been over 80% with a very low dropout rate (<4%). Throughout these trials, we have tested strategies and procedures related to recruitment, retention, data collection, and SPIRIT training, fidelity, and measurement.

In a full-scale multicenter RCT (R01NR011464), we formally tested the efficacy of SPIRIT compared to usual care in preparation for end-of-life decision-making: Measured at 2, 6, and 12 months, primary outcomes were dyad congruence, patient decisional conflict, and surrogate decision-making confidence.[20] We also tested whether SPIRIT reduced post-bereavement distress for surrogates (at 2 wks., 3 and 6 months). For the RCT, 210 dyads of seriously ill dialysis patients and their surrogates from 20 free-standing dialysis facilities (mean age 62, 57% women, 67% African Americans, 96% on hemodialysis) were randomized to SPIRIT or usual care. Intention-to-treat analysis showed that, adjusting for time and baseline values, dyad congruence on goals of care (*OR*=1.89 [95% *CI*, 1.1 to 3.3]; p=.029) and surrogate decision-making confidence (*β*=0.13 [*CI*, 0.01 to 0.24]; *p*=.027) were significantly better in SPIRIT. Patient decisional conflict was significantly lower in SPIRIT at 12 months (*β*=-0.19 [*CI*, -0.33 to -0.04]; *p*=.011). We also created a composite outcome combining dyad congruence and surrogate decision-making confidence because surrogates can feel highly confident even if they misunderstand patients' wishes.[18,34] Thus, to differentiate surrogates who understand the patient's wishes and feel confident in their role from those who do not (i.e., understand the wishes but lack confidence, misunderstand the wishes but feel confident, or neither understand nor feel confident), dyads were grouped as congruent in both scenarios and surrogate decision-making confidence ≥ 3 ("confident" to "very confident"), or not.[18] SPIRIT's effect on the composite outcome was also significant (*OR*=1.82 [95% *CI*, 1.0 to 3.2]; *p*=.041).

Mortality rates between the groups were similar. Among 45 bereaved surrogates, adjusting for time and baseline values, those in the SPIRIT had less anxiety (*β*=-1.13 [*CI*, -2.23 to -0.03]; *p*=.044), depression (*β*=-2.54 [*CI*, -4.34 to -0.74]; *p*=.006), and post-traumatic distress (*β*=-5.75 [*CI*, -10.9 to -0.64]; *p*=.027) than did controls.

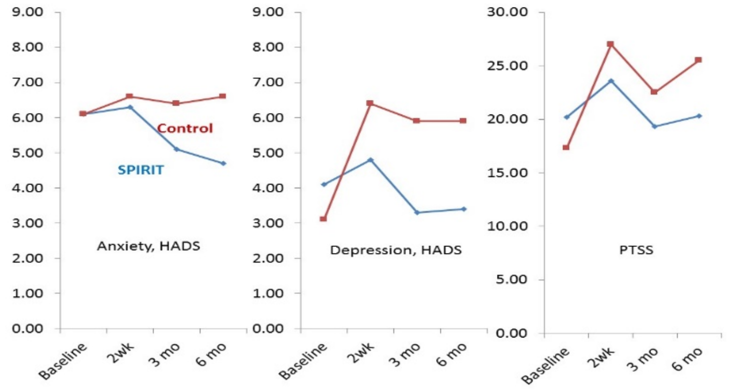

Our qualitative thematic analysis of post-bereavement interviews with surrogates (**Box 1**) helps explain how SPIRIT reduced surrogates' post-bereavement distress.[61] Our data revealed that surrogates reported gaining insight about the life-limiting nature of the patient's illness and that death might be near; notably, no prognostic information was provided during the SPIRIT sessions. An important outcome was that the steps of SPIRIT helped raise prognostic awareness for participants without prognostic estimates having been conveyed.[61]

Box 1. Perceived impact of SPIRIT: Themes

SPIRIT…

a) was an eye-opening experience, acquiring knowledge and understanding of the patient's illness, prognosis, and end-of-life care
b) helped strengthen relationships between patients and surrogates
c) helped surrogates feel prepared during the time leading up to end-of-life decision-making
d) helped surrogates have peace of mind during and after actual end-of-life decision-making

For Supplement Aims:

Adaptation of SPIRIT for dementia and pilot testing: In contrast to people with ESRD, participants in the ongoing parallel trial ("SPIRIT in Dementia," R01AG057714) do not have complex multimorbidity and are expected to live for ~10 years after a diagnosis of dementia. We have completed the iterative process of adaptation for persons with mild to moderate dementia and their surrogates and recently pilot tested the adapted SPIRIT with a sample of 23 PWD (a Montreal Cognitive Assessment [MoCA] score, M=17.7, SD=4.0, range, 13-25; 74% non-Hispanic white; 50% male; mean age=74 years) and their surrogates (65% female, 74% spouse). The PWDs also were assessed for their decision-making

capacity using a screening test, the University of California San Diego Brief Assessment of Capacity to Consent (UBACC),[38] at enrollment (M=13.2, SD=2.1, range 9-16 out of 18). As part of the analysis, using a "quantitizing" technique of qualitative data analysis,[39] we reviewed SPIRIT session transcripts focusing on the values of treatment outcomes and the goals of care discussions, and rated the level of PWD's articulation of end-of-life care preferences on a 3-point scale (from 3=expressed wishes very coherently to 1=unable to express wishes coherently).

Preliminary findings: Of the 23 PWD, 14 PWD (60.9%) had moderate dementia (MoCA=13-17). Of note, all 23 PWD were able to articulate their values and end-of-life wishes somewhat or very coherently; 20 of them expressed their wishes very coherently (rating=3). While MoCA (global cognitive functioning) scores did not differ by the level of articulation of wishes, the UBACC (decision-making capacity) scores did, Kruskal-Wallis H = 5.57, df = 1, p = 0.02, with a mean rank score of 3.50 for a rating of 2 (somewhat coherent, n = 3) and 13.28 for a rating of 3 (very coherent, n = 20).

Our finding that meaningful ACP conversations were possible even for those with moderate dementia and limited decision-making capacity is an important finding because Hirschman et al[40] found that as PWD's cognitive impairment becomes advanced, family members use the best interest standard (what a reasonable person would do) more often than substituted judgement (what my loved one would have wanted). The primary reason for using the best interest standard was that there had been no previous discussion about the PWD's preferences.[41] Our data also show that decision-making capacity may be the more important mental faculty than global cognitive functioning in ACP discussion, particularly for eliciting end-of-life wishes. However, the relationships among cognitive functioning, decision-making capacity, and ability to express wishes need future evaluation. As part of the supplement, we propose to replicate the examination of one's ability to express wishes about end-of-life care in a sample of patients with ESRD plus dementia to assess 1) if these patients have more severe cognitive impairment and how that impacts the ACP conversations, and 2) if complex multimorbidity, i.e., ESRD, impacts the ACP conversation across a range of dementia severity. The supplement also gives us the opportunity to evaluate the adapted SPIRIT with a largely African American sample who are disproportionately represented in conditions with multimorbidity and dementia.

The adapted SPIRIT intervention (SPIRIT-dementia) sessions lasted 100 minutes on average (range, 51-122). The main reasons for the longer duration compared to earlier SPIRIT studies (on average 82 minutes) were that the interventionist was required to: a) speak slowly, b) repeat questions for the PWD, and 3) ask clarifying questions whenever the PWD's response was vague. As is typical with PWD there is slowing of speech and the PWDs often paused to come up with words or collect their thoughts, and the interventionist was prohibited from rushing, interrupting, or finishing the sentence for the PWD. Although the intervention could have been broken down to two sessions, as in previous SPIRIT with other patient populations, we determined that a single session approach would be more appropriate due to PWD's limited or absent short-term memory.

2.3 RISK/BENEFIT ASSESSMENT

2.3.1 KNOWN POTENTIAL RISKS

As the SPIRIT intervention has proven to be safe and efficacious, this Phase III pragmatic trial involves very minimal or low risk. Patient and surrogate participants may experience an emotional reaction (e.g., anxiety) or fatigue during the intervention or data collection. In our previous studies,[17-20] intervention dyads were less apprehensive and more satisfied with the quality of communication than control dyads. It is expected that psychological burden caused by the SPIRIT intervention will be less than or equal to that of usual care.

2.3.2 KNOWN POTENTIAL BENEFITS

Findings from our previous studies indicate benefits of the SPIRIT interventions for participants in the intervention group, including meeting needs to plan for future medical care and sharing values and beliefs. In addition, in our recent study, surrogates in the intervention group perceived the intervention to be highly beneficial during end-of-life decision making for their loved ones and surrogates showed significantly lower post-bereavement distress symptom scores.

2.3.3 ASSESSMENT OF POTENTIAL RISKS AND BENEFITS

As described above, it is expected that psychological burden caused by the SPIRIT intervention will be less than or equal to that of usual care. Previous studies have demonstrated the potential benefits of SPIRIT.

3 OBJECTIVES AND ENDPOINTS

The objective of this multicenter, clinic-level cluster randomized trial is to evaluate the effectiveness of SPIRIT delivered by dialysis care providers as part of routine care in free-standing outpatient dialysis clinics compared to usual care plus delayed SPIRIT implementation. Simultaneously, we will evaluate the implementation of SPIRIT, including sustainability. We will use a Type I effectiveness-implementation hybrid approach[62,63] that combines testing intervention effectiveness and gathering information about implementation of an efficacious intervention in a real world setting. The short-term goal is to generate sufficient evidence to accelerate the integration of SPIRIT into dialysis practice and policy. We will recruit 400 dyads of patients at high risk of death in the next year and their surrogates from 30 dialysis clinics in 4 states.

OBJECTIVES	ENDPOINTS	JUSTIFICATION FOR ENDPOINTS
Primary		
Examine the effectiveness of SPIRIT compared to usual care on preparedness outcomes for end-of-life decision making (defined as dyad congruence on goals of care, patient decisional conflict, and surrogate decision-making confidence) at 2 weeks post-intervention.	Dyad congruence on goals of care (binary);<br>Patient decisional conflict (scale);<br>Surrogate decision-making confidence (scale)<br>Composite outcome (goals-of-care congruence with confident surrogate; binary)	The primary goal of SPIRIT is to prepare the patient and surrogate for end-of-life decision making. The preparedness outcomes will indicate whether or to what extent SPIRIT accomplished the goal.
Secondary		
Examine the effectiveness of SPIRIT and usual care on surrogates' post-bereavement distress	Anxiety symptom score (HAS-anxiety);<br>Depression symptom score (HAS-depression);<br>Post-traumatic distress symptom score (PTSS)	Surrogate prepared by SPIRIT for EOL decision making should experience less post-bereavement distress.
Evaluate the process outcomes of SPIRIT implementation: during the initial and delayed implementation of SPIRIT	Acceptability, fidelity, intervention costs, and sustainability	Descriptive aim to generate data for translation
Tertiary/Exploratory		
Examine the effectiveness of SPIRIT and usual care on EOL treatment intensity	Health care utilization (percentages of patients hospitalized, having ICU admission, and having intensive procedures, and LOS)	To examine whether SPIRIT reduces aggressive EOL treatment

Measurement and data collection time points

Outcome	Measure	Completed by			Time points
		pt	srgt	prov	
**Effectiveness outcome evaluation**					
Preparedness					
Dyad congruence	Goals-of-Care Tool (2 end-of-life scenarios)	√	√		Baseline and 2 wks
Patient decisional conflict	Decisional Conflict Scale (range, 1-5)	√			Baseline and 2 wks
Surrogate decision-making confidence	Decision Making Confidence Scale (range, 0-4)		√		Baseline and 2 wks
Overall preparedness	Preparedness for End-of-Life Decision Making (range, 26-104)	√	√		Baseline and 2 wks
Post-bereavement distress	HADS (each subscale range, 0-21)<br>PTSS-10 (range, 10-70)		√		Baseline and 3 mo. after patient death
End-of-life care intensity	Percentages of patients hospitalized, having ICU admission, and having intensive procedures and length of hospital stay				Medicare claims data after patient death
**Initial implementation process evaluation**					
Care provider acceptability	Dialysis care provider's perceived acceptability (range, 7-28);<br>Semi-structured interview			√	End of the initial implementation
Patient & surrogate acceptability	Patient and surrogate acceptability (range, 10-40 each)	√	√		2 wks
Fidelity	SPIRIT provider's coverage of the SPIRIT components and durations;<br>Brief patient and surrogate survey	√	√	√	SPIRIT provider after each SPIRIT completion<br>Patient and surrogate at 2 wks
Intervention costs	The actual time the care provider spent in carrying out SPIRIT, multiplied by hourly wage (+benefits), plus costs of materials				End of the early implementation
Sustainability	Brief semi-structured interview			√	End of 12 mo.post 9-month follow-up
**Delayed implementation process evaluation**					
Care provider acceptability	Dialysis care provider's perceived acceptability (range, 7-28);<br>Semi-structured interview			√	End of the delayed implementation
Fidelity	SPIRIT provider's coverage of the SPIRIT components durations			√	SPIRIT provider after each SPIRIT completion
Intervention costs	The actual time the care provider spent in carrying out SPIRIT, multiplied by hourly wage (+benefits), plus costs of materials				End of the delayed implementation
**Descriptors and potential covariates**					
Sociodemographics	Sociodemographic Profile	√	√		Baseline for patients and surrogates
				√	End of the initial implementation for clinicians
Clinical characteristics	Medical Profile				Baseline
Clinic-level contextual data	Contextual Data Collection Form				End of each implementation
Usual care	Documented AD completion on the Plan of Care form; presence of DNR, POLST, or MOST (from EMR)				Baseline and 6 mo.

Pt, patient; Srgt, surrogate; Pro, care provider
Data collection highlighted in yellow is performed locally.

For supplement aims:

Measurement and data collection time points

Outcome	Measure	Completed by		Time points
		pt	srgt	
**Estimating the effects of SPIRIT-dementia**				
Preparedness				
Dyad congruence	Goals-of-Care Tool (2 end-of-life scenarios)	√	√	Baseline and 2-3 days
Patient decisional conflict	Decisional Conflict Scale (range, 1-5)	√		Baseline and 2-3 days
Surrogate decision-making confidence	Decision Making Confidence Scale (range, 0-4)		√	Baseline and 2-3 days
Overall preparedness	Preparedness for End-of-Life Decision Making (range, 26-104)	√	√	Baseline and 2-3 days
Post-bereavement distress	HADS (each subscale range, 0-21)		√	Baseline and 1 mo. after patient death
Care decisions	Percentages of patients hospitalized, having ICU admission, and having intensive procedures and length of hospital stay	CMS form 2746		After patient death

4 STUDY DESIGN

4.1 OVERALL DESIGN

We will conduct a dialysis clinic-level cluster randomized trial with two groups, SPIRIT versus usual care followed by delayed SPIRIT. We will recruit 400 dyads of patients on chronic ("prevalent") dialysis who are at high risk of death in the next year and their surrogate decision-makers (total 800 individuals) from 30 free-standing dialysis clinics in 4 states. The primary outcomes are patient and surrogate self-report preparedness for end-of-life decision making. The implementation evaluation data will be obtained throughout the study course. Upon patient death (anticipate 20% of patients; n = ~ 80), we will assess surrogates' post-bereavement distress. Patient participation will end at 9 months (to allow for obtaining Medicare claims data during the study period) or death, whichever occurs first; surrogate participation will end at 9 months or at the completion of 3-month post-death follow-up. If patient is still living at 9 months, we will ask for an extension of follow up period to include 12 more months. Medicare claims data for end-of-life treatment intensity will be obtained quarterly. Clinics assigned to usual care will receive the delayed implementation, SPIRIT, in Year 4, Q1. This is a Phase III trial of an intervention that involves minimal risks and has proven to be efficacious, and thus no interim analysis is planned.

4.2 SCIENTIFIC RATIONALE FOR STUDY DESIGN

We chose cluster over individual randomization for the benefits of increased efficiency and decreased risk of experimental contamination, while recognizing potential loss in statistical precision from the effects of variance inflation.[64] However, cluster effects are usually small and can be controlled in analysis. Cluster randomization is also a favorable design strategy for the Type I hybrid effectiveness-implementation approach to test clinical effectiveness while gathering information on implementation.[62] Hybrid designs are increasingly used to expedite the sequential process, "efficacy to effectiveness to preliminary implementation[63] and are recommended when there is strong evidence of the intervention effects and the intervention is low risk for participants.[62]. To maximize data on the implementation process and sustainability, we chose a delayed intervention design[65] in which clinics are randomized either to implement SPIRIT immediately after randomization (i.e., initial implementation) or to maintain usual care for a comparison condition and then implement the intervention in Year 4 (i.e., delayed implementation). The delayed implementation group will effectively serve as control for effectiveness evaluation. The process outcomes from the initial implementation of SPIRIT will be used to determine if any modifications to

SPIRIT are necessary. We anticipate that we will make clinic-specific adaptations to improve implementation in the delayed implementation phase. Clinics in the delayed implementation phase will be evaluated on the process outcomes, which will provide data on the iterated version of SPIRIT without having to conduct another trial. The delayed implementation is more ethical than not providing SPIRIT at all because its demonstrated efficacy would preclude equipoise.

Virtually no trials are purely pragmatic or explanatory; based on the PRECIS Tool and key characteristics of pragmatic trials,[66,67] the proposed study is more pragmatic than explanatory in the pragmatic-explanatory continuum because it will include diverse patient populations, multiple heterogeneous settings, few inclusion and exclusion criteria, and the comparison condition is a real-world alternative (i.e., usual care), not a placebo. Further, the study is built around normal dialysis care operations as much as possible with flexible study protocols that minimize intrusion in daily work flow at the dialysis facilities. Of direct relevance to dialysis care, the intervention will be implemented by dialysis care providers, such as nurses and social workers.

4.3 JUSTIFICATION FOR INTERVENTION

The SPIRIT intervention is a one-time advance care planning intervention that has been rigorously tested and has demonstrated its efficacy. The details about the intervention, including the rationale, are described above (2.2 Background).

4.4 END OF STUDY DEFINITION

Because the study uses a delayed intervention design to maximize implementation data collection, the end of the study will be the completion of care provider acceptability, fidelity and cost data collection.

5 STUDY POPULATION

5.1 INCLUSION CRITERIA

Patient eligibility criteria:

a) 18 years or older
b) on either hemodialysis or peritoneal dialysis
c) able to understand and speak English.

Surrogate eligibility criteria:

a) 18 years or older (to serve as a surrogate decision-maker, the individual must be an adult)
b) being chosen by the patient.

Roughly 76 providers at the 29 clinics, including all medical directors, nurse managers, social workers, and those who are selected to conduct SPIRIT sessions will participate in the implementation evaluation.

Surrogate eligibility criteria:

a) 18 years or older (to serve as a surrogate decision-maker, the individual must be an adult)
b) being chosen by the patient.

5.2 EXCLUSION CRITERIA

Patient exclusion criteria:

a) lack of an available surrogate,
b) too ill or cognitively impaired to participate based on clinicians' judgment*,
c) already enrolled in hospice.

Surrogate exclusion criterion:

a) Those who cannot complete questionnaires due to physical or cognitive limitations will be excluded.

5.3 LIFESTYLE CONSIDERATIONS

Not applicable

5.4 SCREEN FAILURES

Because patients are approached first at the dialysis center, it is possible that patients provide written consent to participate in the study with the assumption that their surrogates would be willing to participate with them (however, patients cannot complete the baseline without willing surrogates), and then the surrogate actually declines to participate. Because randomization occurs at the clinic level, not the individual level, these cases will be reported under "not eligible" with reason.

5.5 STRATEGIES FOR RECRUITMENT AND RETENTION

Recruitment and Consent procedures:
The dialysis care provider who has been selected by the clinic as one responsible for SPIRIT delivery at each clinic (so-called, "SPIRIT clinician champion") in both groups will generate a list of patients each quarter who meet the inclusion criteria. **Depending on the clinic's structure and workflow, the care provider who determines patients meeting these criteria may not be the same care provider, i.e., SPIRIT clinician champion who is responsible for SPIRIT delivery.**

From this list, the care provider will then assess the patient's willingness to meet with a recruiter from the research team. The recruiter will then approach willing patients during their scheduled dialysis clinic appointment to explain the study purposes and procedures. In a private room, written consent will be obtained from the patient after the study is reviewed and understanding is established. The recruiter will then provide the patient with a study brochure and encourage him/her to talk to the surrogate regarding the study within the next 2-3 days (to avoid a cold call). Several days later, the recruiter will telephone the surrogate to assess his/her willingness to participate. **Using a Surrogate Verbal Consent script and form, the site coordinator will document time and date. Upon this verbal consent, the research staff at the Emory Study Coordination Center will conduct baseline data by phone.** For surrogates in the SPIRIT implementation clinics, the recruiter (at each study site) will schedule the first SPIRIT session to take place at the clinic 2 weeks hence, as is possible.

Recruitment will occur over 24 months beginning at the end of Year 1. We are highly experienced in recruiting seriously ill ESRD patients and their surrogates for research. Consent rates in our previous studies have been consistent at approximately 84% despite the studies' focus on end-of-life, the requirement that both patient and surrogate participate, and long-term follow-up. We expect a similar if not higher consent rate because the study involves a shorter follow-up time and minimal data collection directly from patients and surrogates. Based on our previous studies,[18-20] we conservatively anticipate that 427 patients (20%) will be *eligible and willing* to participate. To reach 400 dyads, we expect to enroll 21-22 dyads per cluster on average. These clinics each accept 10-70 new patients each year (total 700 additional patients), and thus our recruitment goal is readily achievable.

To Maximize Participant Retention, strategies found effective in retaining dyads over 12 months (dropouts, 3.8%) in our efficacy trial[20] will be used: we will

a) obtain two backup contacts from each member of the dyad in the event that we cannot reach them by telephone and mail
b) make confirmation phone calls 2 days prior to each follow-up
c) make monthly check-in calls
d) send holiday cards
e) assign the same data collector whenever possible
f) compensate each member of the dyad with gift cards ($15 at baseline, $15 at 2 weeks for the preparedness outcome assessment; $20 at 3 months for the post-bereavement outcome assessment). Consistent with a pragmatic trial, participants will not be compensated for participating in the intervention. The Emory Study Coordination Center will be responsible for disbursement.

To Maximize Participant Retention, we will

a) obtain two backup contacts from each member of the dyad in the event that we cannot reach them by telephone and mail
b) make confirmation phone calls 2 days prior to the baseline and/or the SPIRIT session (intervention group)
c) make scripted monthly check-in calls
d) send holiday cards
e) assign the same data collector whenever possible
f) provide transportation support ($20) for the SPIRIT session in person at the center (intervention group)
g) compensate each member of the dyad with gift cards ($20 at baseline; $25 at post-intervention follow-up; and surrogates who complete post-bereavement assessment will receive $30 at 1 month after the patient's death).
For supplement cohort study, each member of the dyad will receive an additional $20 at the completion of T3 and $30 at T4.

Recruitment of Care Providers for Implementation data collection, we will obtain a list of dialysis care providers from each dialysis center, who are willing to be contacted by the study staff for participating in implementation related data collection. This data collection is to obtain inputs from the care providers regarding SPIRIT implementation as part of routine dialysis care. Research study staff at the study site will contact care providers on the list using their preferred contact numbers or emails. Verbal Care Provider Informed Consent will be used in consenting process. Survey/Interviews will be scheduled to be conducted over the phone or in person at the convenience of the Care Provider. There will be no compensation for care provider's participation.

6 STUDY INTERVENTION(S)

6.1 STUDY INTERVENTION(S) ADMINISTRATION

6.1.1 STUDY INTERVENTION DESCRIPTION

SPIRIT Intervention:
All care providers responsible for SPIRIT delivery will follow the structured SPIRIT Interview Guide. All sessions will be conducted in a private room in the clinic. The goals of SPIRIT are to assist patients clarify their end-of-life preferences and to help surrogates understand the patient's wishes and prepare for the surrogate role. SPIRIT has two face-to-face sessions with patient and surrogate together. These sessions may occur using teleconferencing software (Zoom) when surrogate decision makers live or work outside a 50-mile radius of patient's clinic. We project the monthly caseload for SPIRIT delivery will be 1-2 dyads per cluster on average.

During the first session (~45 min.), the care provider will assess the patient's and surrogate's cognitive, emotional, and spiritual/religious representations of the patient's illness, prognosis, and end-of-life care. This will allow the care provider to provide individualized information about topics, such as the effectiveness of life-sustaining treatment for people with end-organ failure, and assist the patient to examine his/her values about life-sustaining treatment at the end of life. The care provider will help the surrogate prepare for end-of-life decision-making and for the emotional burden of decision-making by actively involving the surrogate in the discussion. If the surrogate is someone out of the order of the hierarchical compensatory model[71] (e.g., a sibling is chosen instead of a spouse), the care provider will explore potential family conflicts and encourage the dyad to talk with other family members and complete a healthcare power of attorney. A Goals-of-Care document will be completed at the end of the session to indicate the patient's preferences.

A brief second session (~15 min.) will be delivered about **approximately 2 weeks later, if possible**. **For PD patients who scheduled dialysis is varying, the second session of the SPIRIT Intervention can be scheduled to occur approximately 2-4 weeks hence, if possible**. This session is a follow-up to address remaining or new concerns and questions raised after the first session. The patient's Goals-of-Care document will be reviewed and assessed for the need for clarification or correction. The provider will document the patient's end-of-life preferences and the surrogate's name and relationship to the patient in the medical record. If the patient desires a DNR order, POLST, or MOST, the care provider will discuss with the patient's nephrologist and arrange a meeting to complete a treatment order form. We will track completion of these forms.

Usual Care
As required by CMS,[51] written information on ADs is provided to a patient on the first day of dialysis, and a social worker reviews this information with patients and encourages them to complete an AD. This typically takes about 10 minutes. If completed, the presence of an AD is documented on the Plan of Care form. If a patient expresses a desire not to be resuscitated in the dialysis unit, a DNR order is written by a nephrologist and placed in the clinic record. If there is no DNR order in the record, a full code is presumed. A social worker or charge nurse reviews code status and updates it annually. Currently, all four states endorse the physician orders for life-sustaining treatment (POLST) paradigm; NM MOST (medical orders for scope of treatment), NC MOST, PA POLST, VA POST and GA POLST; these forms may be completed to complement ADs. None of the clinics employ routine identification of patients at high risk for death or structured ACP sessions targeting those patients.

6.1.2 DOSING AND ADMINISTRATION

Described above (6.1.1).

6.2 PREPARATION/HANDLING/STORAGE/ACCOUNTABILITY

6.2.1 INTERVENTIONIST TRAINING AND ACCOUNTABILITY

Care Provider Training for SPIRIT
The ideal individual to conduct ACP discussions is still unknown, but we believe it is not the profession per se but rather the willingness and appropriate training that make an individual suitable for conducting the discussions. Each dialysis clinic has identified a care provider designated to conduct SPIRIT sessions (e.g., ANP, RN, MSW). SPIRIT training will occur shortly after randomization for the initial implementation clinics and in early Year 4 for the delayed implementation clinics. The SPIRIT trainers, trained and certified by the PI at the Study Coordination Center (at Emory), will conduct SPIRIT training for all four sites based on the manualized curriculum.

Care provider training will consist of a 1½-day, competency based program that has been used in our previous trials:

- Module 1 (1/2 day) to ensure understanding of end-of-life care issues and communication as key to improving end-of-life care and the Representational Approach (theoretical underpinnings of SPIRIT);
- Module 2 (1/2 day), as skill-base session to gain understanding of the SPIRIT intervention and delivery, including role plays. A 2-week practice period will be scheduled for integration of skills and exploration of additional learning needs; and
- Module 3 (1/2 day) for skill-demonstration and certification. These training sessions will be conducted in collaboration with the Emory Nursing Professional Development Center.

6.2.2 FORMULATION, APPEARANCE, PACKAGING, AND LABELING

Not applicable.

6.2.3 PRODUCT STORAGE AND STABILITY

Not applicable.

6.2.4 PREPARATION

Not applicable.

6.3 MEASURES TO MINIMIZE BIAS: RANDOMIZATION AND BLINDING

Clinics covered by the same care provider who will deliver SPIRIT sessions will be combined to one cluster to avoid risk of contamination; this results in clusters (4 in GA, 3 in NC, 8 in NM, 5 in PA and 8 in VA). Because the numbers of available patients substantially vary across the clusters, to minimize group imbalance clusters will be stratified to three sizes: small (patient census ≤ 52), medium (53-105), and large (≥ 106). We will randomize clusters to either SPIRIT or usual care plus delayed SPIRIT, with randomly permuted blocks (sizes of 2 and 4) within cluster size stratum nested in each state, using a pseudo-random-number generator, by the Study Coordination Center.

Before the study starts, the Study Coordination Center will inform the group assignment for each cluster to the site PIs, with a unique ID consisting of abbreviations for state and cluster size stratum. While the heterogeneity in minority race/ethnicity across clusters improves the study's generalizability, stratification or pairing clusters by race/ethnicity is not feasible because some clinics uniquely serve one race with no comparable clinics within state. Group imbalances on race/ethnicity and other potential confounding factors will be examined and adjusted for in the analyses. The cluster randomized design prevents blinding patient-surrogate dyads and dialysis care providers to group allocation. However, research staff assessing effectiveness outcomes will be blind to group assignment.

6.4 STUDY INTERVENTION COMPLIANCE

To maintain internal validity, certain components of the intervention will be standardized: standardized care provider training using the training modules (described above); Session 1 will be delivered via face-to-face; the SPIRIT Interview Guide will be used during each session to promote consistency and quality of intervention delivery. We will develop a template of procedures related to SPIRIT implementation for each clinic to customize and use as a resource.

To assess fidelity, we will use two independent data sources.

1) The SPIRIT Interview Guide will direct the care provider to document performance data after each patient-surrogate dyad encounter (the data will be entered into REDCap by each site coordinator). The Guide has a checklist of SPIRIT components, including start and finish times and brief self-evaluation.

2) At the 2-week post-intervention follow-up, a research assistant will query patients and surrogates about the SPIRIT sessions using the checklist of SPIRIT components. After the first 50 dyads (~first 4 months) have been seen, clinics with <80% adherence on both data sources will receive feedback and another orientation meeting by the Site PI and the SPIRIT trainer.

We considered replacing clinics with <80% adherence, but this approach would not be consistent with the pragmatic nature of the study to test SPIRIT in a real-world setting. We also considered recording a sample of SPIRIT sessions, but this approach would likely be considered intrusive by the dialysis care providers and is unrealistic in a pragmatic trial.

6.5 CONCOMITANT THERAPY

Not applicable (all patients receive usual care related to advance care planning).

6.5.1 RESCUE MEDICINE

Not applicable.

7 STUDY INTERVENTION DISCONTINUATION AND PARTICIPANT DISCONTINUATION/WITHDRAWAL

7.1 DISCONTINUATION OF STUDY INTERVENTION

SPIRIT is a one-time intervention with two sessions. If patient-surrogate dyads is not able to complete Session I or II, the reason or circumstances (e.g., the patient became too ill) will be documented and reported. Session II cannot be offered without Session I completed; that is, it is possible that a patient-surrogate dyad completes Session I but not Session II. Dyads who never complete Session I and dropout will be replaced by enrolling a new dyad.

7.2 PARTICIPANT DISCONTINUATION/WITHDRAWAL FROM THE STUDY

Participants are free to withdraw from participation in the study at any time upon request.
An investigator may discontinue or withdraw a participant from the study for the following reasons:

- If the participant meets an exclusion criterion that precludes further study participation.

The reason for participant discontinuation or withdrawal from the study will be recorded on the study REDCap. Subjects from the dialysis center randomized to initial SPIRIT who sign the informed consent form but do not receive the study intervention may be replaced. Subjects in the initial SPIRIT who sign the informed consent form and receive the study intervention, and subsequently withdraw, or are withdrawn or discontinued from the study will not be replaced.

7.3 LOST TO FOLLOW-UP

Patients lost to follow-up will not be applicable in this trial since all patients must come to the dialysis center for their treatment and thus can be contacted at the center.

A surrogate participant will be considered lost to follow-up if he or she fails to complete the scheduled 2-week follow-up (after the receipt of the intervention) and is unable to be contacted by the study site staff until the end

of the 9-month follow-up period. Or, a surrogate participant will be considered lost to follow-up if he or she fails to complete the scheduled 3-month follow-up (after the patient's death) and is unable to be contacted by the study site staff.

The following actions must be taken if a participant is determined to be lost to follow-up:

- The site will attempt to contact the participant and reschedule the missed appointment for 4 weeks and ascertain if the participant wishes to continue in the study.
- Before a participant is deemed lost to follow-up, the investigator or designee will make every effort to regain contact with the participant (where possible, 3 telephone calls and, if necessary, a certified letter to the participant's last known mailing address or local equivalent methods, or speak to the patient at the dialysis center). These contact attempts will be documented in the participant's record in REDCap.
- Should the participant continue to be unreachable, he or she will be considered to have withdrawn from the study with a primary reason of lost to follow-up.

8 STUDY ASSESSMENTS AND PROCEDURES

8.1 OUTCOME ASSESSMENTS

Collecting effectiveness outcome data will be centralized; research staff at the Study Coordinating Center will collect the data from patients and surrogates by phone. Telephone-based collection minimizes participants' travel burden. Centralized data collection maintains data collectors being blind to group assignment. The preparedness outcomes will be assessed at 2 weeks post-intervention, as in our R21 trial.[18] We expect roughly 40% of study patients (~160) to die by the 9-month follow-up; deaths are readily identifiable through dialysis clinics and will trigger post-bereavement surveys with surrogates at 3 months and staggered CMS data collection. Post-bereavement assessment at 3 months is based on our efficacy data showing that distress symptoms sharply rose in both groups 2-weeks post-bereavement and then stabilized at 3 months.

Effectiveness Outcomes

Preparedness for end-of-life decision making (measured at baseline and 2 wks post-Session II):

- **Dyad congruence** will be assessed using the Goals-of-Care Tool,[18,20] which includes two scenarios describing medical conditions commonly occurring in ESRD patients. In the first, the patient develops a severe complication and cannot speak for himself/herself; the medical team believed recovery unlikely and continuing life-sustaining treatment, including dialysis, would no longer be beneficial. In the second scenario, the patient develops advanced dementia. Each scenario has three response options: "The goals of care should focus on delaying my death, and thus I want to continue life-sustaining treatment", "The goals of care should focus on my comfort and peace, and thus I do not want life-sustaining treatment, including dialysis", and "I am not sure". Patients and surrogates complete this tool independently and their responses are then compared to determine dyad congruence -- either congruent in both scenarios or incongruent. If both members of the dyad endorse "I am not sure", they are considered incongruent.
- **Patient decisional conflict** will be measured using the 13-item Decisional Conflict Scale (DCS), a validated measure in the context of end-of-life decision making[17]; higher scores indicate greater difficulty in weighing benefits and burdens of life-sustaining treatments and decision making (range 1-5; Cronbach's α = 0.8 – .93[17,18,20,72]).
- **Surrogate decision-making confidence** will be measured using the 5-item Decision Making Confidence (DMC) scale (Cronbach's α = 0.81-0.90[18,34]) on which higher scores reflect greater comfort in performing as a surrogate (range 0, not confident at all-4, very confident). DMC is a self-report of a surrogate's confidence in: knowledge of the patient's wishes, ability to make treatment decisions even in a highly stressful situation, ability to seek information about risks and benefits of medical choices, ability to handle

unwanted pressure from others, and ability to communicate with health care providers about the patient's wishes.

- **Composite outcome**: We will also create a composite outcome combining dyad congruence and surrogate DMC to differentiate surrogates who understand the patient's wishes and feel confident in their role from those who don't (understand the wishes but lack confidence, misunderstand the wishes but feel confident, neither understand nor feel confident).[18,34]
- We will also assess **the overall preparedness for end-of-life decision making** using the 26-item investigator-developed measure. The measure assesses the level of preparedness for end-of-life decision making in the cognitive, emotional, and behavioral dimensions on a 4-point scale (4=strongly agree to 1=strongly disagree) with higher scores indicating higher levels of preparedness. Patient and surrogate each will complete this measure separately.

Implementation Process Outcomes

For the initial SPIRIT intervention, after the final dyads have completed the SPIRIT sessions (~Year 3, Q1), care providers will complete a survey and an interview.

- **Care provider acceptability** will be evaluated using the 7-item Care Providers' Perceived Acceptability Survey to assess their perceptions about SPIRIT implementation, including the time required, impact on interactions with patients, and whether they would recommend SPIRIT to other clinics.[80] Response options range from 1=strongly disagree to 4=strongly agree; higher scores indicate greater acceptability.
- **A brief (~15 min.) semi-structured interviews (face-to-face or by telephone; audio-recorded) will obtain providers' perspectives** on whether the implementation of SPIRIT is compatible with their setting and the workflow, it's perceived utility, their willingness to continue using it after the study, factors influencing implementation (barriers, logistical constraints, and facilitators), and suggestions for improvement.
- **Patient and surrogate acceptability** will be assessed using the 10-item ACP Acceptability Questionnaire developed from our previous trial,[24] Participants are asked how strongly they agree or disagree (4 to 1) with statements about their experience with SPIRIT sessions, including duration, interactions with the care provider, level of comfort and satisfaction. Higher scores indicate greater acceptability. Each patient and surrogate will complete this survey at the 2-week post-intervention follow-up.
- **Fidelity/adherence** will be assessed using two independent data sources, (a) the SPIRIT Interview Guide, Checklist, and self-evaluation completed by the care provider after each SPIRIT session and (b) patient and surrogate responses to the SPIRIT components coverage during the 2-week post-intervention follow-up as described above. The number of SPIRIT components covered, the minutes required for the care provider to complete the sessions, the number of dyads who complete SPIRIT sessions, and the number of incomplete or interrupted sessions will be aggregated.
- **Intervention cost**s will be estimated based on the actual time the care provider spent in carrying out SPIRIT, multiplied by hourly wage (+benefits), plus costs of materials. Overhead (e.g., facility) costs and research staff's time will not be included.
- We define **sustainability** as the extent to which a newly implemented intervention is maintained within a service setting's ongoing, stable operations.[81] In Y4, Q4, we will conduct a brief interview with SPIRIT care providers to ask about sustainability: (a) Is SPIRIT on-going (and at what frequency)? (b) What components of the SPIRIT protocol have been retained? and (c) Has SPIRIT implementation been evaluated at the clinic level? If so, how?[82]

For the delayed SPIRIT implementation, the process evaluation will involve care providers only since patient and surrogate study participation will have ended at the end of 9-month follow-up or patient death. Care provider acceptability, fidelity, and intervention costs will be determined as described above.

End-of-life treatment intensity (upon patient death)

We will link study data to publicly available USRDS data. The USRDS captures inpatient and outpatient Medicare claims on all treated U.S. ESRD patients and releases these data annually free of charge. Medicare claims data are

the most complete and reliable source of data on end-of-life care intensity because patients on dialysis are Medicare beneficiaries. The USRDS data are far superior to that which could be obtained through individual hospitals because data collection at the latter is extremely difficult and highly likely to result in missing data. From the inpatient and outpatient claims data, we will obtain dates and attributed causes of death; dates of hospital, skilled nursing facility, and hospice admissions and discharges; dates of outpatient encounters (including dialysis sessions and ED visits); and diagnostic and procedure codes for all inpatient and outpatient encounters. Thus, we will be able to determine hospitalization, ICU days, days hospitalized, use of intensive procedure, (dis)continuation of dialysis (also from CMS Form 2746), and hospice use during the final month of life. We will consider the following to be intensive procedures: mechanical ventilation, feeding tube placement, dialysis, and cardiopulmonary resuscitation.[22] These intensive procedures will be identified using HCPCS and ICD-9-CM codes (e.g., ICD-9 codes for intubation and mechanical ventilation: 96.04, 96.05, 96.7X).[83]

Descriptors and Potential Covariates

- Patients and surrogates will complete a **Sociodemographic Profile** which includes age, gender, race and ethnicity, type of relationship between patient and surrogate, marital status, religious affiliation, education, household income, previous end-of-life decision-making experience, and previous participation in an ACP discussion or AD completion.
- To describe the sample, **the patient's clinical characteristics**, including dialysis modality, years on dialysis, comorbid conditions will be abstracted from the patient's EMR.
- **Care providers' sociodemographic data** will include age, gender, race and ethnicity, education, and years of practice.
- At baseline, we will collect **clinic-level contextual data**[84] that could facilitate understanding study results, including staffing, patient census, rural-urban status,[85-89] palliative care and hospice availability, and proximity of hospitals.

8.2 SAFETY AND OTHER ASSESSMENTS

SPIRIT is a one-time psychoeducational intervention. The study involves very low risk and the potential risk may include fatigue or emotional upset during the session.

Fatigue

As part of outcome assessment, HADS and PTSS-10 will be completed. Although these measures are not diagnostic tools, if a surrogate's HADS-Depression is high (≥ 15), the data collector will ask the surrogate if she/he is aware of the mood state and encourage to speak to his/her primary care provider. If a surrogate expresses suicidal ideation, the data collector will immediately notify the local site coordinator who will confer with the Site PI. This Site PI will determine if referral to the local mental health clinic (during hours) or the 24-hour Emergency Psychiatry Service (after hours) for evaluation of mental health or emergency intervention is necessary.

8.3 ADVERSE EVENTS AND SERIOUS ADVERSE EVENTS

8.3.1 DEFINITION OF ADVERSE EVENTS (AE)

SPIRIT is a one-time psychoeducational intervention. The study involves very low risk and the potential risk may include fatigue or emotional upset during the session, neither of which is a "medical occurrence". SPIRIT has been extensively tested in previous trials and no safety concerns have ever arisen. The present trial is to generate data to accelerate translation of the intervention into clinical practice.

8.3.2 DEFINITION OF SERIOUS ADVERSE EVENTS (SAE)

The trial targets ESRD patients on dialysis who already have serious life-threatening medical conditions and are likely to die within a year (by clinician's judgment). SPIRIT is an advance care planning intervention to prepare these patients and their surrogates for end-of-life decision making. SPIRIT's safety and beneficial effects (e.g., reducing psychological distress) have been consistently demonstrated. Participants' deaths or hospitalizations (or other events described above) during the trial are expected (and needed to answer the scientific questions) and will occur as part of the illness course. These events will not be considered as SAE in this trial. However, any participant's death will be reported to IRB through annual progress report and included in the NIH annual progress report.

8.3.3 CLASSIFICATION OF AN ADVERSE EVENT

8.3.3.1 SEVERITY OF EVENT

Not applicable.

8.3.3.2 RELATIONSHIP TO STUDY INTERVENTION

Not applicable.

8.3.3.3 EXPECTEDNESS

There are no known expected adverse reactions. SPIRIT has been tested in 5 RCTs with various patient populations with serious chronic conditions and in 3 different regions and settings. Although possible adverse reactions to the intervention may include fatigue or emotional distress during the intervention session, no such reactions have been observed in the previous studies. Thus, these reactions are very unlikely to occur and will be considered "unexpected." Intervention sessions will stop if any of these events occurs.

8.3.4 TIME PERIOD AND FREQUENCY FOR EVENT ASSESSMENT AND FOLLOW-UP

Not applicable.

8.3.5 ADVERSE EVENT REPORTING

The possible adverse reactions (fatigue, emotional distress during the intervention session), if ever occurs, will be tracked (documented in the study REDCap) and the aggregated numbers will be reported at the upcoming biannual DSMB meeting.

Any disease-related events (DREs) common in the study population (e.g., expected) such as death will not be reported per the standard process of reporting but will be monitored so that surrogates' post-bereavement outcomes and end-of-life treatment intensity data (Medicare claims data) can be collected timely. However, any participant's death will be reported to IRB through annual progress report and included in the NIH annual progress report.

8.3.6 SERIOUS ADVERSE EVENT REPORTING

Not applicable.

8.3.7 REPORTING EVENTS TO PARTICIPANTS

Not applicable.

8.3.8 EVENTS OF SPECIAL INTEREST

Not applicable.

8.3.9 REPORTING OF PREGNANCY

Not applicable.

8.4 SUBJECT SAFETY

Any unexpected problem related to the Research that negatively affects the rights, safety or welfare of subjects and is not described as such in the materials describing risks associated with the study.

8.4.1 DEFINITION OF UNANTICIPATED PROBLEMS (UP)

Unanticipated problems are defined by DHHS 45 CFR part 46 as any incident, experience, or outcome that meets all of the following criteria:

- unexpected, in terms of nature, severity, or frequency, given (a) the research procedures that are described in the protocol-related documents, such as the IRB-approved research protocol and informed consent document; and (b) the characteristics of the study population;
- related or possibly related to participation in the research (in this guidance document, possibly related means there is a reasonable possibility that the incident, experience, or outcome may have been caused by the procedures involved in the research);
- suggests that the research places participants or others at a greater risk of harm (including physical, psychological, economic, or social harm) than was previously known or recognized.

It will be extremely unlikely in this study that the events of fatigue and emotional distress would meet the all of the criteria above.

8.4.2 UNANTICIPATED PROBLEM REPORTING

If we encounter any adverse event that meets the definition above and that is related to the intervention, the PI will notify the Emory IRB and NINR Program Official and the DSMB within 24 hours of the event being reported to the PI. The expedited report will be followed by a detailed, written SAE report as soon as possible.

8.4.3 REPORTING UNANTICIPATED PROBLEMS TO PARTICIPANTS

Not applicable.

9 STATISTICAL CONSIDERATIONS

9.1 STATISTICAL HYPOTHESES

- Primary Endpoint(s):
  1) The number of SPIRIT dyads who are congruent on goals of care at 2 weeks post-intervention will be significantly higher than that of control dyads.
  2) Patient decisional conflict scores at 2 weeks post-intervention will be significantly lower than those of control dyads.
  3) Surrogate decision making confidence (DMC) scores at 2 weeks post-intervention will be significantly higher than those of control dyads.
  4) The number of SPIRIT dyads who are congruent on goals of care and confident surrogate (DMC=3 or higher) at 2 weeks post-intervention will be significantly higher than that of control dyads.

- Secondary Endpoint(s):
  1) HAD-anxiety scores in SPIRIT surrogates will be significantly lower than those of control surrogates at 3 months after the patient's death.
  2) HAD-depression scores in SPIRIT surrogates will be significantly lower than those of control surrogates at 3 months after the patient's death.
  3) PTSS-10 scores in SPIRIT surrogates will be significantly lower than those of control surrogates at 3 months after the patient's death.

- Exploratory: Among patients who have died,
  1) The percentage of patients hospitalized during the final month of life in the SPIRIT group will be significantly lower than that in the control group.
  2) The percentage of patients admitted to an ICU during the final month of life in the SPIRIT group will be significantly lower than that in the control group.
  3) The percentage of patients having intensive procedures during the final month of life in the SPIRIT group will be significantly lower than that in the control group.
  4) The length of hospital stay during the final month of life in the SPIRIT group will be significantly shorter than that in the control group.

9.2 SAMPLE SIZE DETERMINATION

We have an adequate sample size to detect clinically meaningful differences between SPIRIT and usual care for our primary outcomes. Statistical power is based on a random effects models: a generalized linear mixed model for binary outcomes (e,g., dyad congruence) and a linear mixed model for continuous outcomes (e.g., patient decisional conflict).

We conducted a simulation study to estimate power with 2-sided significance level alpha=.05, corrected for anticipated dropout and potential intraclass correlations (ICCs) ranging .01-.04, based on ICCs observed in our prior work. To estimate the power more

Power for comparing SPIRIT and usual care with 19 clusters

**Aim 1**	Percent or min. difference	*OR* or variance	ICC[a]	Estimated power
Dyad congruence	48%	*OR*=2.0	0.02<br>0.04	0.92<br>0.84
Patient DCS	*d*=0.24	0.23	0.01<br>0.10	0.98<br>0.81
Surrogate DMC	*d*=0.22	0.20	0.01<br>0.10	0.99<br>0.80
Composite outcome	47%	*OR*=2.0	0.02	0.93
**Aim 3**				
Anxiety	*d*=1.35	7.0	0.01<br>0.03	0.84<br>0.80
Depression	*d*=1.60	10.0	0.01<br>0.03	0.85<br>0.79
PTSS-10	*d*=4.75	90.0	0.01<br>0.03	0.85<br>0.78

[a] observed ICCs in our prior work; *d*=1/2 *SD*.
DCS, decisional conflict scale; DMC, decision-making confidence.

conservatively, we used the effect sizes observed at 2 months[20] rather than those at 2 weeks,[18] which are larger (e.g., for the composite outcome, *OR*=1.8 at 2 months, *OR*=4.4 at 2 weeks). The overall power to detect clinically meaningful differences is excellent for the primary outcomes and good for the post-death outcomes.

9.3 POPULATIONS FOR ANALYSES

Patient-surrogate dyads will be the primary unit of analysis; all analyses will be intent to treat with all available data from all participants.

9.4 STATISTICAL ANALYSES

9.4.1 GENERAL APPROACH

The preliminary analysis will include summarizing variables with standard descriptive statistics and graphical displays or frequency tables. Distributional assumptions will be assessed and the data will be transformed as necessary. Clinic characteristics (e.g., rural-urban status) will be compared using χ^2 tests for categorical variables and t-tests for continuous variables. We will compare SPIRIT and usual care participants on baseline characteristics (e.g., age, race/ethnicity) to explore possible between-group differences using generalized estimating equation (GEE) methods, accounting for the observed correlation within the same cluster.[90,91] In our previous work SPIRIT has no effect on patient mortality[20]; however, we will compare survival time between SPIRIT and usual care using Cox proportional hazards models, adjusted for cluster effects. If imbalanced, we will consider adjustment for the group difference.

9.4.2 ANALYSIS OF THE PRIMARY ENDPOINT(S)

SPIRIT effectiveness on the preparedness outcomes: Dyad congruence and the composite outcome (binary variables) will be analyzed by fitting a generalized mixed effects model for each, where the binary outcome is modeled in terms of a logit link[90] with both a random intercept and random slope to control for variation within and between subjects and clusters. The model also includes the intervention SPIRIT, cluster size and their interaction. For patient DCS and surrogate DMC scores, we will replace the logit link by the identity link with an additional error term. These models will allow us to examine whether SPIRIT was superior to usual care in the primary outcomes at 2 weeks and whether the effect of SPIRIT varies by cluster size. The analysis will be adjusted for potential covariates, such as race/ethnicity, and rural-urban status, in the model, including interaction between treatment and race/ethnicity.

9.4.3 ANALYSIS OF THE SECONDARY ENDPOINT(S)

Analysis of secondary endpoints are not dependent on findings of primary endpoints.

SPIRIT effectiveness on surrogates' post-bereavement psychological distress: We will use the same approach as in the analysis of the primary endpoints to compare anxiety, depression, and post-traumatic distress symptoms in SPIRIT vs usual care among surrogates of patients who die during the initial implementation and 9-month (or extended) follow-up.

Implementation process outcomes: Quantitative data on acceptability, fidelity, and costs of SPIRIT will be summarized using descriptive statistics. SPIRIT will be determined to be acceptable to care providers and patients and surrogates if over 75% of responses exceed an average summative score ≥ 3 (of 4) on the acceptability measures. Cost estimates will be used to ascertain resources needed to implement or replicate SPIRIT in the future.[92] We will also explore the relationships of these quantitative data with characteristics of settings and

stakeholders. Transcripts of acceptability interviews will be transferred to ATLAS.ti for analysis. Content analysis techniques[93] will be used without preconceived categories.[94] Open coding will be applied[95] and disagreements on coding will be resolved by consensus. We will examine the data for patterns or differences in themes between those with acceptability scores >=3 and scores <3 and setting characteristics. This analysis will be facilitated by creating matrixes that organize textual and numeric data for comparing and contrasting[96] so that what contributed to acceptability scores that are positive and less than positive may be identified. We will use a similar approach, content analysis, to evaluate the sustainability data.

9.4.4 SAFETY ANALYSES

Not applicable.

9.4.5 BASELINE DESCRIPTIVE STATISTICS

See 9.4.1 General Approach.

9.4.6 PLANNED INTERIM ANALYSES

Not applicable.

9.4.7 SUB-GROUP ANALYSES

In order to evaluate whether the SPIRIT has differential effects according to demographic factors such as age, sex, race/ethnicity we consider the same generalized mixed effects model in the context of protocol design, mentioned in Section 9.4.1. Our approach is first to consider each factor one at a time. We will fit the same generalized mixed model by additionally including a subject level factor (e.g., race) and its' interaction with the SPIRIT. If the interaction is significant the analysis will indicate that SPIRIT effect changes according to race. We will report p-values and importantly, standard errors and confidence intervals by recognizing the fact that we may not have power to detect significant interaction effects for examining all demographic factor combinations.

9.4.8 TABULATION OF INDIVIDUAL PARTICIPANT DATA

Individual participant data will be listed by measure and time point.

9.4.9 EXPLORATORY ANALYSES

SPIRIT effectiveness on end-of-life treatment intensity: Among patients who die during the initial SPIRIT implementation and follow-up, percentages of patients hospitalized, having ICU admission, having intensive procedures and length of hospital stay in the final month of life will be summarized using descriptive statistics, 95% CIs, and graphical displays. The exploratory examination of SPIRIT's effectiveness on improving these outcomes, we will use the same analytic approach as in the analysis of the primary endpoints (See 9.4.2).

10 SUPPORTING DOCUMENTATION AND OPERATIONAL CONSIDERATIONS

10.1 REGULATORY, ETHICAL, AND STUDY OVERSIGHT CONSIDERATIONS

10.1.1 INFORMED CONSENT PROCESS

10.1.1.1 CONSENT/ASSENT AND OTHER INFORMATIONAL DOCUMENTS PROVIDED TO PARTICIPANTS

Consent forms describing in detail the study intervention, study procedures, and risks are given to the participant and written documentation of informed consent is required prior to starting intervention/administering study intervention. The following consent materials are submitted with this protocol.

- Patient Consent Form-SPIRIT
- Patient Consent Form-Usual Care
- Surrogate Verbal Consent Form –SPIRIT
- Surrogate Verbal Consent Form – Usual Care
- Care Provider Verbal Consent Form
- VERBAL Consent Addendum – Patient
- VERBAL Consent Addendum - Surrogate

10.1.1.2 CONSENT PROCEDURES AND DOCUMENTATION

Participating dialysis centers/clinics will be randomized first (see the study flow diagram). The care team member who has been selected by the clinic as a care provider responsible for SPIRIT delivery at each clinic in both groups (e.g., nurse or social worker) will generate a list of patients each quarter based on the inclusion criteria. This staff member may differ from clinic to clinic depending on who is qualified, comfortable and able to assess each potential participant. The medical director of the dialysis unit (and/or the attending physician as required) approves the list if required by the dialysis center. From this list, one of the care providers will then assess the patient's willingness to meet with a recruiter from the research team. The recruiter (e.g., site coordinator) will then approach willing patients during their scheduled dialysis clinic appointment.

Consent forms have been Institutional Review Board (IRB)-approved and the participant will be asked to read and review the document. In a private room, a verbal explanation will be provided in terms suited to the patient's comprehension of the purposes, procedures, and potential risks of the study and of their rights as research participants. Patient participants will have the opportunity to carefully review the written consent form (including HIPAA authorization from) and ask questions prior to signing. The patient participants should have the opportunity to discuss the study with their family or surrogates or think about it prior to agreeing to participate. The patient participant will sign the informed consent document prior to any procedures being done specifically for the study. Patient participants must be informed that participation is voluntary and that they may withdraw from the study at any time, without prejudice. A copy of the informed consent document will be given to the participants for their records. The informed consent process will be conducted and documented in the source document (including the date), and the form signed, before the participant undergoes any study-specific procedures. The rights and welfare of the participants will be protected by emphasizing to them that the quality of their medical care will not be adversely affected if they decline to participate in this study. The recruiter will then provide the patient participant with a study brochure and encourage him/her to talk to the surrogate regarding the study within the next 2-3 days (to avoid a cold call).

Several days later, the recruiter will telephone the potential surrogate participant to assess his/her willingness to participate. Following the Surrogate Verbal Consent Form, a verbal explanation will be provided in terms suited to the surrogate's comprehension of the purposes, procedures, and potential risks of the study and of their rights as research participants. Upon the surrogate's verbal consent using the IRB approved script/form, the research staff at the Emory Study Coordination Center will schedule and conduct a baseline data collection session. All surrogates will provide verbal consent only with a waiver of written consent and a Surrogate Verbal Consent form will be signed by the recruiter.

10.1.2 STUDY DISCONTINUATION AND CLOSURE

It is very unlikely that this study may be suspended or prematurely terminated sine the SPIRIT intervention has been extensively tested, including its safety and efficacy. Also, this study aim includes collecting implementation data and no planned interim analysis and stopping rules. Nonetheless, if suspension or termination occurs, written notification, documenting the reason for study suspension or termination, will be provided by the suspending or terminating party to study participants, investigator, dialysis organizations, and regulatory authorities. If the study is terminated or suspended, the Principal Investigator (PI) will promptly inform study participants, the Institutional Review Board (IRB), and sponsor and will provide the reason(s) for the termination or suspension. Study participants will be contacted, as applicable, and be informed of changes to study appointment schedule.

Circumstances that may warrant termination or suspension include, but are not limited to:

- Determination of unexpected, significant, or unacceptable risk to participants
- Insufficient compliance to protocol requirements

Study may resume once concerns about safety, protocol compliance, and data quality are addressed, and satisfy the sponsor and IRB.

10.1.3 CONFIDENTIALITY AND PRIVACY

Participant confidentiality and privacy is strictly held in trust by the participating investigators, their staff, and the sponsor. The study documentation, data, and all other information generated will be held in strict confidence. No information concerning the study or the data will be released to any unauthorized third party without prior written approval of the sponsor.

All research activities will be conducted in as private a setting as possible.

The study monitor, other authorized representatives of the sponsor, representatives of the Institutional Review Board (IRB), regulatory agencies may inspect all documents and records required to be maintained by the investigator, including but not limited to, medical records (office, clinic), for the participants in this study. The clinical study site will permit access to such records.

The study participant's contact information will be securely stored in REDCap study database for internal use during the study. At the end of the study, all paper records will continue to be kept in a secure location for as long a period as dictated by the reviewing IRB, Institutional policies, or sponsor requirements.

Study participant research data, which is for purposes of statistical analysis and scientific reporting, will be directly entered into and stored in REDCap study database. Individual participants and their research data will be identified by a unique study identification number. All information collected during the study will be secured and password protected. At the end of the study, all study databases will be de-identified and archived at the Emory Study Coordination Center.

10.1.4 FUTURE USE OF STORED SPECIMENS AND DATA

Data collected for this study will be analyzed and stored at the Emory Study Coordination Center. After the study is completed, the de-identified, archived data will be transmitted to and stored in the REDCap study archive for

use by other researchers including those outside of the study. When the study is completed, access to study data will be provided through the Emory Study Coordination Center.

10.1.5 KEY ROLES AND STUDY GOVERNANCE

Study Coordination Center	Principal Investigator	Project Director
	Mi-Kyung Song, PhD, RN<br>Professor<br>Emory University, School of Nursing<br>1520 Clifton Rd NE, Atlanta, GA 30322<br>404-727-3134<br>mi-kyung.song@emory.edu	Mary Laszlo<br>Project Manager<br>Emory School of Nursing<br>404-727-2882<br>Mary.laszlo@emory.edu
**Study Site**	**Site PI**	**Site Coordinator**
Emory University	Mi-Kyung Song	Mary Laszlo<br>Project Manager
University of North Carolina at Chapel Hill	Abhijit Kshirsagar, MD, MPH<br>Associate Professor<br>Chief Medical Director, UNC Dialysis Care<br>UNC Kidney Center<br>CB 7155 Burnett-Wormack<br>Chapel Hill, NC 27599<br>919-445-2684<br>abhijit_kshirsagar@med.unc.edu	
University of Pittsburgh	Manisha Jhamb, MD, MPH<br>Assistant Professor<br>UPMC Presbyterian 200 Lothrop St<br>Suite C1100, Rm C1103<br>Pittsburgh, PA 15213<br>412-647-7062<br>jhambm@upmc.edu	
University of New Mexico	Mark Unruh, MD, MS<br>Professor and Chair<br>UNM, Internal Medicine<br>DoIM MSC10-5550<br>1 University of New Mexico<br>Albuquerque, NM 87131<br>505-272-0407<br>MLUnruh@salud.unm.edu	
University of Virginia	Maureen Metzger, RN, PhD<br>Associate Professor<br>University of Virginia School of Nursing<br>PO Box 800782<br>5038 McLeod Hall<br>Charlottesville, VA 22908-0782<br>434-924-0112<br>Mjm9cd@virginia.edu<br><br>Emaad Abdel-Rahman, MD PhD, FASN (Co-Investigator)<br>Prof. Internal Medicine/Nephrology<br>Division of Nephrology<br>UVA	

	PO Box 800133<br>Charlottesville, VA 22908<br>434-243-2671<br>ea6n@virginia.edu	

Other study co-investigators:

Laura Plantinga, PhD<br>Assistant Professor<br>Emory University School of Medicine<br>Renal Division<br>laura.plantinga@emory.edu	Janice I.P. Lea, MD, MSc<br>Professor of Medicine<br>Chief Medical Director Emory Dialysis<br>Clinical Specialist in Hypertension<br>Emory University School of Medicine<br>Renal Division<br>550 Peachtree St. 7th floor, MOT<br>Atlanta, GA 30308<br>404-686-5038<br>jlea@emory.edu	Amita Manatunga, PhD<br>Professor<br>Rollins School of Public Health<br>Emory University<br>1518 Clifton Rd NE<br>Atlanta, GA 30322<br>404-727-1309<br>amanatu@sph.emory.edu
Sandra Ward, PhD, RN, Professor Emerita<br>University of Wisconsin-Madison<br>608-257-0119<br>sward@wisc.edu		

Study organization and coordination

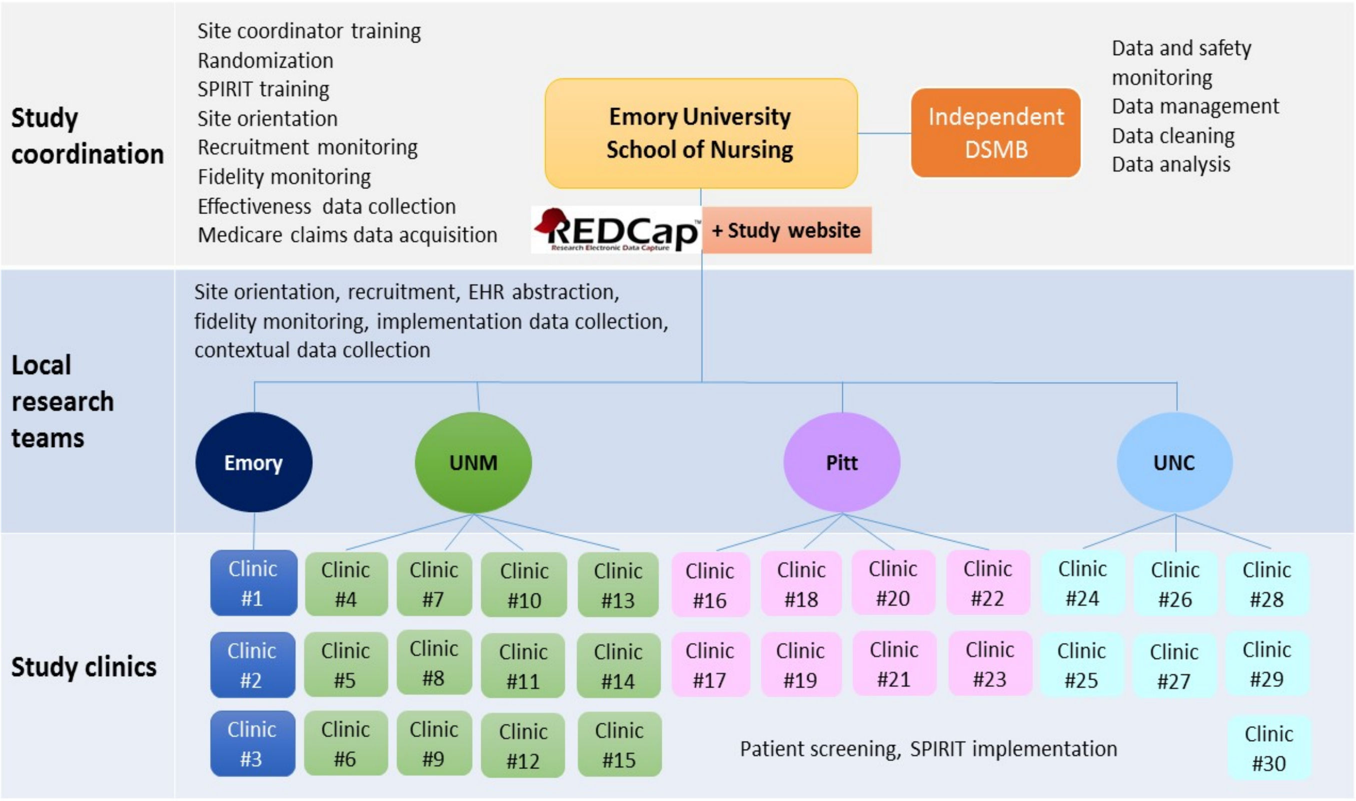

10.1.6 SAFETY OVERSIGHT

Safety oversight will be under the direction of a Data and Safety Monitoring Board (DSMB) composed of 5 individuals with the appropriate expertise, including dialysis care, advance care planning, palliative care, clinical

trials, intervention testing, and biostatistics. Members of the DSMB are independent from the study conduct and free of conflict of interest (see signed disclosure forms).

The DSMB will meet bi-annually to evaluate the study progress, including assessment of data quality, timeliness, participant recruitment, rates of eligibility and ineligibility across study clinics and sites/states, accrual and retention, participant risk versus benefit, performance of trial sites, patient deaths, and any adverse events or care providers' concerns that can affect study outcome. Consistent with prior end-of-life communication interventions, including our prior trials, we do not expect that the intervention will alter mortality rates. Nonetheless, this will be one of the measures monitored by the DSMB. The DSMB will compare recruitment rate and sample characteristics to assumptions used for power calculations. The DSMB will monitor the design factor, intra-cluster correlation coefficient, to assess whether the study will have expected power. Formal interim analyses are not planned for several reasons: (a) the SPIRIT efficacy has been established, (b) risks associated with SPIRIT are very minimal, (c) the control condition is usual care, not a placebo, and (d) the proposed study also aims to gather implementation data, including sustainability, that are critical for widespread implementation. Finally, the DSMB will not monitor fidelity/adherence data and make recommendations for ways to improve adherence because of the pragmatic nature of the proposed trial (i.e., seeking real world answers). The DMSB will operate under the rules of an approved charter that will be written and reviewed at the organizational meeting of the DSMB.

10.1.7 CLINICAL MONITORING

Clinical site monitoring is conducted to ensure that the rights and well-being of trial participants are protected, that the reported trial data are accurate, complete, and verifiable, and that the conduct of the trial is in compliance with the currently approved protocol/amendment(s), with International Conference on Harmonisation Good Clinical Practice (ICH GCP), and with applicable regulatory requirement(s).

All study data will be directly entered into REDCap. The data entry forms in REDCap will be set up such that out-of-range values are not accepted, which will minimize data entry error. Although HADS (anxiety and depression symptom scales) and PTSS-10 (post-traumatic distress symptom screening) are not diagnostic tools, when a surrogate's HADS score is abnormal (Depression score ≥15), the REDCap data entry form will flag the research staff to notify the surrogate that the local research staff member may confer with the Site PI so that the participant can be referred to a local mental health if necessary.

- The PI and Project Director will conduct centralized monitoring quarterly throughout the study. A random review of 10% primary endpoint data and secondary endpoint data (HADS and PTSS-10) will be performed. A monitoring report will be generated at completion of review and will be shared with the study site teams.
- Independent audits will not be conducted as this trial does not collect clinical data after baseline (as descriptors).

10.1.8 QUALITY ASSURANCE AND QUALITY CONTROL

We will use centralized training of research staff for recruitment and data collection activities as appropriate. The PI and Project Director will train staff in all study procedures. All project staff will complete university sponsored research integrity training, including modules on the protection of human subjects, HIPAA, and Good Clinical Practice. All roles, responsibilities, and a protocol with scripted subject contacts will be clearly delineated in the study Standard Operating Procedures (SOPs). These SOPs will be accessible via REDCap.

Data collectors/recruiters will attend a competency based, one-day training session that the PI and Project Director will convene. Following a demonstration by the PI or Project Director on how to recruit participants and obtain informed consent, the recruiters will be expected to perform three satisfactory practice recruitment sessions before actual performance. The final practice sessions will be audio- or video-recorded and reviewed for adherence to the protocol. After demonstrating satisfactory performance of consenting sessions, the recruiters will be authorized to recruit and enroll participants.

Training for data collection will include scripted data collection techniques with special attention to assessing participant fatigue or discomfort during the data collection session. Data collectors will conduct a series of three practice baseline and follow-up data collections using volunteers. After demonstrating satisfactory performance on data collection, they will be authorized to perform data collection activities with enrolled participants. They will also need to demonstrate completeness of data collection activities using REDCap.

Each study site will perform internal quality management of study conduct, data collection, documentation and completion. In general the following strategies will be employed:

- Use of data collection and data entry SOP
- Before ending the data collection session, review the data entry form in REDCap for any missing data
- Each data collector signs his/her work
- Audit research staff members' performance (e.g., consenting and data collection) Biannually.

We will also employ systematic checking of data quality: The project director at the Emory Study Coordination Center will run quality control checks on the database quarterly; any missing data or data anomalies will be reported to the PI and communicated to the site(s) for clarification/resolution.

The PI/Dr. Song will convene weekly meetings with staff to review progress, subject accrual, and any unanticipated problems at the Emory site. Site PIs will do the same at their respective study sites. The weekly progress information will be aggregated for reports and presented at bi-monthly or monthly all study sites meetings.

To ensure compliance with the monitoring plan and reporting requirements across study sites, the PI/Dr. Song at the Study Coordination Center will convene a videoconference (using Adobe Connect or Zoom) with the study investigators, project director, SPIRIT trainer, and site coordinators monthly. At these meetings the investigators will assess study performance related to subject recruitment across the study sites (at least 1 dyad per month in a small cluster and 3 dyads in a larger cluster), review the quality of the data, and discuss any adverse events.

10.1.9 DATA HANDLING AND RECORD KEEPING

10.1.9.1 DATA COLLECTION AND MANAGEMENT RESPONSIBILITIES

The School of Nursing at Emory University, the Emory site, will be the Study Coordination Center and will maintain close contact with every entity within the study and will monitor study activities. All study sites will use a common study web-portal using the Research Electronic Data Capture (REDCap) created and managed in Emory SON. Each site will create and update study participants' data through REDCap. During data entry, automated checks will be performed that will immediately flag problematic data (e.g., missing, out of range, inconsistent), allowing for the sites to address any discrepant data promptly thus increasing data quality. Data entered into the web-based form are immediately stored in a study database and tracked through a journaling process where they are accessible for review by the study team. Social Security numbers are entered directly into REDCap and not listed on paper forms. Suspicious data can be flagged through a query management system, and automated alerts provided to the sites. A complete audit trail is stored for each database modification. Any discrepant data identified through

analytic manipulations will be communicated to the sites. Once all queries have been resolved and the database has been deemed “clean”, it will be officially locked. All permissions to make changes (append, delete, modify or update) to the database by the sites will be removed at that time.

Record keeping and data collection (recruitment data, patient medical record review at enrollment, clinic-contextual data, SPIRIT implementation data) are the responsibilities of the research staff at the site under the supervision of the site investigator. The investigator is responsible for ensuring the accuracy, completeness, legibility (if hardcopies of worksheets are used), and timeliness of the data reported.

10.1.9.2 STUDY RECORDS RETENTION

All study’s written records will be stored in a locked cabinet for 5 years. Study data will be de-identified and shared with future researchers per written request and IRB approval (Resource and Data Sharing Plans).

10.1.10 PROTOCOL DEVIATIONS

A protocol deviation is any noncompliance with the clinical trial protocol, International Conference on Harmonisation Good Clinical Practice (ICH GCP), or Manual of Procedures (MOP) requirements. The noncompliance may be either on the part of the participant, the investigator, or the study site staff. As a result of deviations, corrective actions are to be developed by the site and implemented promptly.

It is the responsibility of the site investigator to use continuous vigilance to identify and report deviations within 7 working days of identification of the protocol deviation. See Protocol Deviation/Violation Report Form and the related SOP. All deviations will be addressed in study source documents, reported to the Study Coordination Center. The site investigator is responsible for knowing and adhering to the reviewing IRB requirements.

10.1.11 PUBLICATION AND DATA SHARING POLICY

This study will be conducted in accordance with the following publication and data sharing policies and regulations:
National Institutes of Health (NIH) Public Access Policy, which ensures that the public has access to the published results of NIH funded research. It requires scientists to submit final peer-reviewed journal manuscripts that arise from NIH funds to the digital archive PubMed Central upon acceptance for publication.
This study will comply with the NIH Data Sharing Policy and Policy on the Dissemination of NIH-Funded Clinical Trial Information and the Clinical Trials Registration and Results Information Submission rule. As such, this trial has been registered at ClinicalTrials.gov, and results information from this trial will be submitted to ClinicalTrials.gov. In addition, every attempt will be made to publish results in peer-reviewed journals.

Authorship determination: Authorship confers credit and has important academic, social, and financial implications. Authorship also implies responsibility and accountability for published work. We will follow the recommendations by the International Committee of Medical Journal Editors (ICMJE) to determine authorship (vs. non-author contributors). http://www.icmje.org/recommendations/browse/roles-and-responsibilities/defining-the-role-of-authors-and-contributors.html
Authorship will be based on the following 4 criteria:

1. Substantial contributions to the conception or design of the work or the acquisition, analysis, or interpretation of data for the work; AND
2. Drafting the work or revision it *critically for important intellectual content* (simply participating in writing or technical editing of the manuscript is insufficient for authorship); AND
3. Final approval of the version to be published; AND

4. Agreement to be accountable for all aspects of the work in ensuring that questions related to the accuracy or integrity of any part of the work are appropriately investigated and resolved.

Those who do not meet all 4 of the above criteria will be acknowledged as non-author contributors.

Data sharing:
We will make the final data from the study, including a codebook, available to researchers after acceptance for publication of the main findings from the final dataset. The final data will be a complete and cleaned data set free of identifiers. We will make the research data available to users with a data-sharing agreement that includes: (1) a commitment to using the data only for research purposes, (2) a commitment to securing the data using appropriate computer technology, (3) a commitment to destroying the data after analyses are completed and not redistributing to third parties, and (4) IRB approval and clear research questions. Data request and sharing procedures, data request forms, and a data-sharing agreement will be accessible through the website of Center for Nursing Excellence in Palliative Care, Nell Hodgson Woodruff School of Nursing. The requester will be able to download final dataset and codebook. Also, care providers or administrators who wish to use the SPIRIT intervention in their practice and care setting can place a request through the Center's website and will be able to download the SPIRIT intervention manual.

10.1.12 CONFLICT OF INTEREST POLICY

Any actual conflict of interest of persons who have a role in the design, conduct, analysis, publication, or any aspect of this trial will be disclosed and managed. Furthermore, persons who have a perceived conflict of interest will be required to have such conflicts managed in a way that is appropriate to their participation in the design and conduct of this trial. The study leadership in conjunction with the NINR has established policies and procedures for all study group members to disclose all conflicts of interest and will establish a mechanism for the management of all reported dualities of interest.

11 REFERENCES

1. United States Renal Data System. 2015 USRDS annual data report: Epidemiology of kidney disease in the United States. Bethesda, MD: National Institutes of Health, National Institute of Diabetes and Digestive and Kidney Diseases, 2015.

2. Renal Physicians Association. Shared decision-making in the appropriate initiation of and withdrawal from dialysis: Clinical practice guideline. 2nd ed. Rockville, MD: Renal Physicians Association, 2010.

3. Kolarik RC, Arnold RM, Fischer GS, Tulsky JA. Objectives for advance care planning. J Palliat Med 2002;5:697-704.

4. Tulsky JA. Beyond advance directives: importance of communication skills at the end of life. JAMA 2005;294:359-65.

5. Institute of Medicine. Dying in America: Improving quality and honoring individual preferences near the end of life. Washington, D.C.: The National Academy of Sciences, 2014.

6. Davison SN. Facilitating advance care planning for patients with end-stage renal disease: The patient perspectives. Clinical Journal of the Amedican Society of Nephrology 2006;1:1023-1028.

7. Goff SL, Eneanya ND, Feinberg R, Germain MJ, Marr L, Berzoff J, Cohen LM, Unruh M. Advance Care Planning: A Qualitative Study of Dialysis Patients and Families. Clin J Am Soc Nephrol 2015;10:390-400.

8. Braun UK, Beyth RJ, Ford ME, McCullough LB. Voices of African American, Caucasian, and Hispanic surrogates on the burdens of end-of-life decision making. Journal of General Internal Medicine 2008;23:267-74.

9. Cherlin E, Fried T, Prigerson HG, Schulman-Green D, Johnson-Hurzeler R, Bradley EH. Communication between physicians and family caregivers about care at the end of life: when do discussions occur and what is said? J Palliat Med 2005;8:1176-85.

10. Cohen LM, Germain MJ, Woods AL, Mirot A, Burleson JA. The family perspective of ESRD deaths. American Journal of Kidney Diseases 2005;45:154-61.

11. Cohen LM, Poppel DM, Cohn GM, Reiter GS. A very good death: measuring quality of dying in end-stage renal disease. J Palliat Med 2001;4:167-72.

12. Hebert RS, Dang Q, Schulz R. Preparedness for the death of a loved one and mental health in bereaved caregivers of patients with dementia: findings from the REACH study. J Palliat Med 2006;9:683-93.

13. Hebert RS, Schulz R, Copeland VC, Arnold RM. Preparing Family Caregivers for Death and Bereavement. Insights from Caregivers of Terminally Ill Patients. Journal of Pain and Symptom Management 2009;37:3-12.

14. Wendler D, Rid A. Systematic review: the effect on surrogates of making treatment decisions for others. Annals of Internal Medicine 2011;154:336-46.

15. Donovan HS, Ward S. A representational approach to patient education. J Nurs Scholarsh 2001;33:211-6.

16. Donovan HS, Ward SE, Song MK, Heidrich SM, Gunnarsdottir S, Phillips CM. An update on the representational approach to patient education. J Nurs Scholarsh 2007;39:259-65.

17. Song MK, Kirchhoff KT, Douglas J, Ward SE, Hammes BJ. A randomized, controlled trial to improve advance care planning among patients undergoing cardiac surgery. Medical Care 2005;43:1049-1053.

18. Song MK, Ward SE, Happ MB, Piraino B, Donovan HS, Shields AM, Connolly MC. Randomized controlled trial of SPIRIT: An effective approach to preparing African American dialysis patients and families for end-of-life. Research in Nursing & Health 2009;32:260-273.

19. Song MK, Donovan HD, Piraino B, Choi J, Bernardini J, Verosky D, Ward SE. Effects of an intervention to improve communication about end-of-life care among African Americans with chronic kidney disease. Applied Nursing Research 2010;23:65-72.

20. Song MK, Ward SE, Fine JP, Hanson LC, Lin FC, Hladik GA, Hamilton JB, Bridgman JC. Advance care planning and end-of-life decision making in dialysis: A randomized controlled trial targeting patients and their surrogates. American Journal of Kidney Diseases 2015;66:813-22.

21. Goodman DC, Esty AR, Fisher ES, Chaning CH. Trends and variation in end-of-life care for Medicare beneficiaries with severe chronic illness: A report of the Darthmouth Atlas Project. Lebanon, NH: Dartmouth Institute for Health Policy & Clinical Practice, 2011.

22. Wong SP, Kreuter W, O'Hare AM. Treatment intensity at the end of life in older adults receiving long-term dialysis. Archives of Internal Medicine 2012;172:661-3; discussion 663-4.

23. Cohen LM, Germain MJ, Poppel DM. Practical considerations in dialysis withdrawal: "to have that option is a blessing". JAMA 2003;289:2113-9.

24. Holley JL, Hines SC, Glover JJ, Babrow AS, Badzek LA, Moss AH. Failure of advance care planning to elicit patients' preferences for withdrawal from dialysis. American Journal of Kidney Diseases 1999;33:688-93.

25. Calvin AO. Haemodialysis patients and end-of-life decisions: a theory of personal preservation. Journal of Advanced Nursing 2004;46:558-66.

26. Weisbord SD, Carmody SS, Bruns FJ, Rotondi AJ, Cohen LM, Zeidel ML, Arnold RM. Symptom burden, quality of life, advance care planning and the potential value of palliative care in severely ill haemodialysis patients. Nephrology, Dialysis, Transplantation 2003;18:1345-52.

27. Davison SN. End-of-life care preferences and needs: perceptions of patients with chronic kidney disease. Clin J Am Soc Nephrol 2010;5:195-204.

28. Singer PA, Thiel EC, Naylor CD, Richardson RM, Llewellyn-Thomas H, Goldstein M, Saiphoo C, Uldall PR, Kim D, Mendelssohn DC. Life-sustaining treatment preferences of hemodialysis patients: implications for advance directives. Journal of the American Society of Nephrology 1995;6:1410-7.

29. Song MK, Ward SE, Hanson LC, Metzger M, Kim S. Determining consistency of surrogate decisions and end-of-life care received with patient goals-of-care preferences. Journal of Palliative Medicine 2016;19:610-6.

30. Pruchno RA, Lemay EP, Jr., Feild L, Levinsky NG. Predictors of patient treatment preferences and spouse substituted judgments: the case of dialysis continuation. Medical Decision Making 2006;26:112-21.

31. Pruchno RA, Lemay EP, Jr., Feild L, Levinsky NG. Spouse as health care proxy for dialysis patients: whose preferences matter? Gerontologist 2005;45:812-9.

32. Houts RM, Smucker WD, Jacobson JA, Ditto PH, Danks JH. Predicting elderly outpatients' life-sustaining treatment preferences over time: The majority rules. Medical Decision Making 2002;22:39-52.

33. Sulmasy DP, Haller K, Terry PB. More talk, less paper: Predicting the accuracy of substituted judgments. American Journal of Medicine 1994;96:432-8.

34. Song MK, Ward SE, Lin FC. End-of-life decision-making confidence in surrogates of African-American dialysis patients is overly optimistic. J Palliat Med 2012;15:412-7.

35. Badger JM. Factors That Enable or Complicate End-of-Life Transitions in Critical Care. American Journal of Critical Care 2005;14:513-21.

36. Boyle DK, Miller PA, Forbes-Thompson SA. Communication and end-of-life care in the intensive care unit: patient, family, and clinician outcomes. Critical Care Nursing Quarterly 2005;28:302-16.

37. Swigart V, Lidz C, Butterworth V, Arnold R. Letting go: family willingness to forgo life support. Heart and Lung 1996;25:483-94.

38. Hardin SB, Yusufaly YA. Difficult end-of-life treatment decisions: do other factors trump advance directives? Archives of Internal Medicine 2004;164:1531-3.

39. Abbott KH, Sago JG, Breen CM, Abernethy AP, Tulsky JA. Families looking back: one year after discussion of withdrawal or withholding of life-sustaining support. Critical Care Medicine 2001;29:197-201.

40. Azoulay E, Pochard F, Kentish-Barnes N, Chevret S, Aboab J, Adrie C, Annane D, Bleichner G, Bollaert PE, Darmon M, Fassier T, Galliot R, Garrouste-Orgeas M, Goulenok C, Goldgran-Toledano D, Hayon J, Jourdain M, Kaidomar M, Laplace C, Larche J, Liotier J, Papazian L, Poisson C, Reignier J, Saidi F, Schlemmer B. Risk

of post-traumatic stress symptoms in family members of intensive care unit patients. American Journal of Respiratory and Critical Care Medicine 2005;171:987-94.

41. Curtis JR, Engelberg RA, Wenrich MD, Shannon SE, Treece PD, Rubenfeld GD. Missed Opportunities during Family Conferences about End-of-Life Care in the Intensive Care Unit. American Journal of Respiratory and Critical Care Medicine 2005;171:844-9.

42. Siegel MD, Hayes E, Vanderwerker LC, Loseth DB, Prigerson HG. Psychiatric illness in the next of kin of patients who die in the intensive care unit. Critical Care Medicine 2008;36:1722-8.

43. Tilden VP, Tolle SW, Nelson CA, Fields J. Family decision-making to withdraw life-sustaining treatments from hospitalized patients. Nursing Research 2001;50:105-15.

44. Baggs JG, Schmitt MH. End-of-life decisions in adult intensive care: current research base 158 and directions for the future. Nursing Outlook 2000;48:158-64.

45. Hansen L, Archbold PG, Stewart BJ. Role strain and ease in decision-making to withdraw or withhold life support for elderly relatives. J Nurs Scholarsh 2004;36:233-8.

46. Tilden VP, Tolle SW, Garland MJ, Nelson CA. Decisions about life-sustaining treatment. Impact of physicians' behaviors on the family. Archives of Internal Medicine 1995;155:633-8.

47. Jacob DA. Family members' experiences with decision making for incompetent patients in the ICU: a qualitative study. American Journal of Critical Care 1998;7:30-6.

48. Jeffers BR. The surrogate's experience during treatment decision-making. Medsurg Nursing 1998;7:357-63.

49. Shiozaki M, Hirai K, Dohke R, Morita T, Miyashita M, Sato K, Tsuneto S, Shima Y, Uchitomi Y. Measuring the regret of bereaved family members regarding the decision to admit cancer patients to palliative care units. Psycho-Oncology 2008;17:926-31.

50. Wright AA, Zhang B, Ray A, Mack JW, Trice E, Balboni T, Mitchell SL, Jackson VA, Block SD, Maciejewski PK, Prigerson HG. Associations between end-of-life discussions, patient mental health, medical care near death, and caregiver bereavement adjustment. JAMA 2008;300:1665-73.

51. Centers for Medicare and Medicaid Services. Medicare and Medicaid Programs; Conditions for Coverage for End-Stage Renal Disease Facilities; Final Rule. In: Services DoHaH, editor, 2008.

52. Centers for Medicare & Medicaid Services. 42 CFR Parts 405, 410. 411, 414, 425, and 495 [CMS-1631-FC] Medicare Program; Revisions to Payment Polocies under the Physician Fee Sschedule and Other Revisions to Part B for CY 2016. 2015.

53. Fagerlin A, Schneider CE. Enough. The failure of the living will. Hastings Center Report 2004;34:30-42.

54. Leventhal H, Nerenz D, Steele DS. Illness representations and coping with health threats. In: Baum A, Singer JE, editors. Handbook of psychology and health. New York: Erlbaum, 1984:221-252.

55. Posner G, Strike K, Hewson P, Gertzog W. Accommodation of a scientific conception: Toward a theory of conceptual change. Science Education 1982;66:211-227.

56. Kleinman A, Eisenberg L, Good B. Culture, illness, and care: clinical lessons from anthropologic and cross-cultural research. Annals of Internal Medicine 1978;88:251-8.

57. Kleinman A, Mendelsohn E. Systems of medical knowledge: a comparative approach. Journal of Medicine and Philosophy 1978;3:314-30.

58. Hewson M. Patient education through teaching for conceptual change. Journal of General Internal Medicine 1993;8:393-8.

59. Hewson P, Hewson M. The role of conceptual conflict in conceptual change and the design of instruction. Instructional Science 1984;13:1-13.

60. Song MK, Ward SE. Making visible a theory-guided advance care planning intervention. J Nurs Scholarsh 2015;47:389-96.

61. Song MK, Metzger M, Ward SE. Process and impact of an advance care planning intervention evaluated by bereaved surrogate decision-makers of dialysis patients. Palliative Medicine 2016.

62. Curran GM, Bauer M, Mittman B, Pyne JM, Stetler C. Effectiveness-implementation hybrid designs: combining elements of clinical effectiveness and implementation research to enhance public health impact. Medical Care 2012;50:217-26.

63. NIH Health Care Systems Research Collaboratory. Pragmatic Trials eBook. In rethinking Clinical Trials: A Living Texbook of Pragmatic Clinical Trials.

64. Donner A, Klar N. Pitfalls of and controversies in cluster randomization trials. American Journal of Public Health 2004;94:416-22.

65. Krist AH, Glenn BA, Glasgow RE, Balasubramanian BA, Chambers DA, Fernandez ME, Heurtin-Roberts S, Kessler R, Ory MG, Phillips SM, Ritzwoller DP, Roby DH, Rodriguez HP, Sabo RT, Sheinfeld Gorin SN, Stange KC, Group MS. Designing a valid randomized pragmatic primary care implementation trial: the my own health report (MOHR) project. Implement Sci 2013;8:73.

66. Loudon K, Treweek S, Sullivan F, Donnan P, Thorpe KE, Zwarenstein M. The PRECIS-2 tool: designing trials that are fit for purpose. BMJ 2015;350:h2147.

67. Thorpe KE, Zwarenstein M, Oxman AD, Treweek S, Furberg CD, Altman DG, Tunis S, Bergel E, Harvey I, Magid DJ, Chalkidou K. A pragmatic-explanatory continuum indicator summary (PRECIS): a tool to help trial designers. Journal of Clinical Epidemiology 2009;62:464-75.

68. Lynn J, Schuster JL, Kabcenell A. Improving care for the end of life: A sourncebook for health care managers and clinicians. New York, NY: Oxford University Press, 2000.

69. Moss AH, Ganjoo J, Sharma S, Gansor J, Senft S, Weaner B, Dalton C, MacKay K, Pellegrino B, Anantharaman P, Schmidt R. Utility of the "surprise" question to identify dialysis patients with high mortality. Clin J Am Soc Nephrol 2008;3:1379-84.

70. Cohen LM, Ruthazer R, Moss AH, Germain MJ. Predicting six-month mortality for patients who are on maintenance hemodialysis. Clin J Am Soc Nephrol 2010;5:72-9.

71. Carr D, Khodyakov D. Health care proxies: whom do young old adults choose and why? Journal of Health and Social Behavior 2007;48:180-94.

72. Song MK, Sereika SM. An evaluation of the Decisional Conflict Scale for measuring the quality of end-of-life decision making. Patient Education and Counseling 2006;61:397-404.

73. Lautrette A, Darmon M, Megarbane B, Joly LM, Chevret S, Adrie C, Barnoud D, Bleichner G, Bruel C, Choukroun G, Curtis JR, Fieux F, Galliot R, Garrouste-Orgeas M, Georges H, Goldgran-Toledano D, Jourdain M, Loubert G, Reignier J, Saidi F, Souweine B, Vincent F, Barnes NK, Pochard F, Schlemmer B, Azoulay E. A communication strategy and brochure for relatives of patients dying in the ICU. New England Journal of Medicine 2007;356:469-78.

74. Zigmond AS, Snaith RP. The hospital anxiety and depression scale. Acta Psychiatrica Scandinavica 1983;67:361-70.

75. Lowe B, Spitzer RL, Grafe K, Kroenke K, Quenter A, Zipfel S, Buchholz C, Witte S, Herzog W. Comparative validity of three screening questionnaires for DSM-IV depressive disorders and physicians' diagnoses. Journal of Affective Disorders 2004;78:131-40.

76. Eid J, Thayer JF, Johnsen BH. Measuring post-traumatic stress: a psychometric evaluation of symptom--and coping questionnaires based on a Norwegian sample. Scandinavian Journal of Psychology 1999;40:101-8.

77. Weisaeth L. Torture of a Norwegian ship's crew. Stress reactions, coping, and psychiatric aftereffects. In: Wilson JP, Raphael B, editors. International Handbook of Traumatic Stress Syndromes. London: Plenum Press, 1993.

78. Schelling G, Stoll C, Haller M, Briegel J, Manert W, Hummel T, Lenhart A, Heyduck M, Polasek J, Meier M, Preuss U, Bullinger M, Schuffel W, Peter K. Health-related quality of life and posttraumatic stress disorder in survivors of the acute respiratory distress syndrome. Crit Care Med 1998;26:651-9.

79. Johansen VA, Wahl AK, Eilertsen DE, Weisaeth L. Prevalence and predictors of post-traumatic stress disorder (PTSD) in physically injured victims of non-domestic violence. A longitudinal study. Soc Psychiatry Psychiatr Epidemiol 2007;42:583-93.

80. McLean KA. Healthcare provider acceptability of a behavioral intervention to promote adherence. Department of Psychology: University of Miami, 2013:434.

81. Glasgow RE, McKay HG, Piette JD, Reynolds KD. The RE-AIM framework for evaluating interventions: what can it tell us about approaches to chronic illness management? Patient Education and Counseling 2001;44:119-27.

82. Wiltsey Stirman S, Kimberly J, Cook N, Calloway A, Castro F, Charns M. The sustainability of new programs and innovations: a review of the empirical literature and recommendations for future research. Implement Sci 2012;7:17.

83. Barnato AE, Farrell MH, Chang CC, Lave JR, Roberts MS, Angus DC. Development and validation of hospital "end-of-life" treatment intensity measures. Medical Care 2009;47:1098-105.

84. Stange KC, Glasgow RE. Considering and Reporting Important Contextual Factors in Research on the Patient-Centered Medical Home. In: Quality AfHRa, editor. Rockville, MD: Agency for Healthcare Research and Quality, 2013.

85. United States Department of Agriculture. Urban influence code. 2013.

86. Gessert CE, Haller IV, Johnson BP. Regional variation in care at the end of life: discontinuation of dialysis. BMC Geriatr 2013;13:39.

87. Watanabe-Galloway S, Zhang W, Watkins K, Islam KM, Nayar P, Boilesen E, Lander L, Wang H, Qiu F. Quality of end-of-life care among rural Medicare beneficiaries with colorectal cancer. Journal of Rural Health 2014;30:397-405.

88. Lavergne MR, Lethbridge L, Johnston G, Henderson D, D'Intino AF, McIntyre P. Examining palliative care program use and place of death in rural and urban contexts: a Canadian population-based study using linked data. Rural Remote Health 2015;15:3134.

89. Wang H, Qiu F, Boilesen E, Nayar P, Lander L, Watkins K, Watanabe-Galloway S. Rural-Urban Differences in Costs of End-of-Life Care for Elderly Cancer Patients in the United States. Journal of Rural Health 2015.

90. Verbeke G, Molenberghs G. Linear mized models for longitudinal data. New York: Springer, 2009.

91. Molenberghs G, Verbeke G. Models for discrete longitudinal data. New York: Springer, 2005.

92. Ritzwoller DP, Sukhanova A, Gaglio B, Glasgow RE. Costing behavioral interventions: a practical guide to enhance translation. Annals of Behavioral Medicine 2009;37:218-27.

93. Hsieh HF, Shannon SE. Three approaches to qualitative content analysis. Qualitative Health Research 2005;15:1277-88.

94. Kondracki NL, Wellman NS, Amundson DR. Content analysis: review of methods and their applications in nutrition education. J Nutr Educ Behav 2002;34:224-30.

95. Miles MB, Huberman AM. Qualitative Data Analysis: An expanded sourcebook. 2nd ed ed. Thousand Oaks, CA: Sage Publishing, 1994.

96. Braun V, Clarke V. Using thematic analysis in psychology. Qualitative Research in Psychology 2006;3:77-101.
