# Supplementary material for: Effectiveness of an Advance Care Planning Intervention in Adults Receiving Dialysis and Their Families: A Cluster Randomized Clinical Trial
Source: JAMA Netw Open. 2024 Jan 30;7(1):e2351511. doi: 10.1001/jamanetworkopen.2023.51511 (PMC10828909; doi:10.1001/jamanetworkopen.2023.51511)
Supplement: Supplement 2. — Data Sharing Statement [file jamanetwopen-e2351511-s002.pdf]

Data Sharing Statement

Song. Effectiveness of an Advance Care Planning Intervention in Adults Receiving Dialysis and Their Families. *JAMA Netw Open*. Published January 30, 2024.
doi:10.1001/jamanetworkopen.2023.51511

Data
Data available: Yes
Data types: Deidentified participant data
How to access data: Data request should be made to mi-kyung.song@emory.edu
When available: With publication

Supporting Documents
Document types: None

Additional Information
Who can access the data: Researchers whose proposed use of the data has been approved
Types of analyses: As specified in the agreement
Mechanisms of data availability: With a signed data access agreement and review of the proposal to be sure the data can support the question(s)
